# Antimicrobial activity of α-mangostin against *Staphylococcus* species from companion animals *in vitro* and therapeutic potential of α-mangostin in skin diseases caused by *S. pseudintermedius*

**DOI:** 10.3389/fcimb.2023.1203663

**Published:** 2023-05-25

**Authors:** Seong Yong Park, Jung Hwa Lee, Seo Yeon Ko, Nayeong Kim, Seong Yeop Kim, Je Chul Lee

**Affiliations:** Department of Microbiology, School of Medicine, Kyungpook National University, Daegu, Republic of Korea

**Keywords:** *Staphylococcus* species, companion animals, α-Mangostin, antimicrobial activity, MAP domain-containing protein

## Abstract

Antimicrobial resistance in *Staphylococcus* species from companion animals is becoming increasingly prevalent worldwide. *S. pseudintermedius* is a leading cause of skin infections in companion animals. α-mangostin (α-MG) exhibits various pharmacological activities, including antimicrobial activity against G (+) bacteria. This study investigated the antimicrobial activity of α-MG against clinical isolates of *Staphylococcus* species from companion animals and assessed the therapeutic potential of α-MG in skin diseases induced by *S. pseudintermedius* in a murine model. Furthermore, the action mechanisms of α-MG against *S. pseudintermedius* were investigated. α-MG exhibited antimicrobial activity against clinical isolates of five different *Staphylococcus* species from skin diseases of companion animals *in vitro*, but not G (-) bacteria. α-MG specifically interacted with the major histocompatibility complex II analogous protein (MAP) domain-containing protein located in the cytoplasmic membrane of *S. pseudintermedius* via hydroxyl groups at C-3 and C-6. Pretreatment of *S. pseudintermedius* with anti-MAP domain-containing protein polyclonal serum significantly reduced the antimicrobial activity of α-MG. The sub-minimum inhibitory concentration of α-MG differentially regulated 194 genes, especially metabolic pathway and virulence determinants, in *S. pseudintermedius*. α-MG in pluronic lecithin organogel significantly reduced the bacterial number, partially restored the epidermal barrier, and suppressed the expression of cytokine genes associated with pro-inflammatory, Th1, Th2, and Th17 in skin lesions induced by *S. pseudintermedius* in a murine model. Thus, α-MG is a potential therapeutic candidate for treating skin diseases caused by *Staphylococcus* species in companion animals.

## Introduction

Antimicrobial resistance (AMR) is a major threat to public health and veterinary medicine (World Health Organization, 2014; World Organisation for Animal Health, 2016). AMR in bacterial pathogens derived from livestock is increasing due to the misuse and overuse of antimicrobial agents for treatment, metaphylaxis, and growth promotion (Van Boeckel et al., 2015). In addition, AMR in bacterial pathogens from companion animals is an emerging problem that increases the risk to public health because antimicrobial agents important in human medicine are commonly used to treat bacterial infections in companion animals (Johnston, 1998; Joosten et al., 2020). Livestock and companion animals are potential reservoirs of resistant bacteria and AMR genes (Joosten et al., 2020; Ibekwe et al., 2023). AMR genes or resistant bacteria can be transferred between companion animals and humans by close contact, food chain, or environment (Bennani et al., 2020).

The most common reason to visit veterinary hospitals for disease treatment is skin and subcutaneous infections such as dermatitis and pyoderma in Korea (Rural Development Administration of Korea, 2018). *Staphylococcus* species, including *S. pseudintermedius, S. schleiferi*, and *S. felis*, are most commonly isolated from skin lesions of companion animals (Devriese et al., 2009; Palomino-Farfan et al., 2021; Cavana et al., 2023). *S. pseudintermedius* is the leading cause of skin, ear, and postoperative bacterial infections in dogs and cats (Devriese et al., 2009; Bannoehr and Guardabassi, 2012; Pompilio et al., 2015). Pyoderma caused by *Staphylococcus* species in companion animals is commonly treated with antimicrobial agents. However, multidrug resistance was found in 75% of *S. pseudintermedius* isolates and 39% of *S. schleiferi* isolates from companion dogs and cats (Animal and Plant Quarantine Agency, 2020). Notably, resistance to methicillin was found in 40% and 27% of *S. schleiferi* and *S. pseudintermedius* isolates, respectively (Animal and Plant Quarantine Agency, 2020). Regarding the prevalence of AMR in *Staphylococcus* species from skin lesions of companion animals, it is necessary to develop antibiotic alternatives to control bacterial skin infections in companion dogs and cats.

Natural products are an important source of antimicrobial agents or serve as templates for synthetic drugs. A wide range of structurally unique secondary metabolites from plants is also important for developing new antimicrobial agents (Zaynab et al., 2018). Mangosteen (*Garcinia mangostana* Linn) is a tropical fruit tree cultivated in Southeast Asia for centuries. Mangosteen pericarps have been used as a traditional medicine for treating cystitis, diarrhea, abdominal pain, eczema, fever, itching, and other skin diseases (Pedraza-Chaverri et al., 2008; Zaynab et al., 2018; Gunter et al., 2020). The bioactive secondary metabolites of mangosteen are xanthone derivatives that possess a potent pharmacological activity (Jung et al., 2006; Akao et al., 2008; Gunter et al., 2020; Liu et al., 2022). α-mangostin (α-MG) is a major xanthone derivative that exhibits broad-spectrum pharmaceutical activities, including antibacterial, antifungal, antiparasitic, anti-inflammatory, antitumor, antidiabetic, antioxidant, and antiobesity (Iinuma et al., 1996; Ibrahim et al., 2016). α-MG has demonstrated antimicrobial activity against G (+) bacteria such as *S. aureus*, *S. epidermidis*, *Streptococcus pyogenes*, *S. mutans*, and vancomycin-resistant enterococci (Sakagami et al., 2005; Nguyen et al., 2014; Sivaranjani et al., 2017; Felix et al., 2022). α-MG interacts with the cytoplasmic membrane of *S. aureus* and disrupts the cytoplasmic membrane integrity, resulting in a rapid bactericidal action (Koh et al., 2013; Felix et al., 2022), but the exact action mechanisms of α-MG in killing *Staphylococcus* species have not been characterized yet. Moreover, the antimicrobial activity of α-MG against *Staphylococcus* species commonly isolated from companion animals has not been determined. This study was aimed to investigate the antimicrobial activity of α-MG against clinical isolates of *Staphylococcus* species from skin diseases of companion dogs and cats and elucidate the action mechanisms of α-MG in killing *S. pseudintermedius*. Finally, the therapeutic potential of α-MG in skin diseases of companion dogs and cats induced by *S. pseudintermedius* was assessed in a murine model.

## Materials and methods

### Bacterial strains

Ten reference bacterial strains, including eight different *Staphylococcus* species, *Escherichia coli*, and *Pseudomonas aeruginosa*, were used in this study (**Table 1**). Seven and three reference strains were obtained from the American Type Culture Collection (ATCC; Manassas, VA, USA) and Korean Collection for Type Cultures (KCTC; Daejeon, Korea), respectively. A total of 277 clinical isolates, including *S. pseudintermedius* (n = 80), *S. schleiferi* (n = 64), *S. felis* (n = 51), *S. epidermidis* (n = 27), *S. aureus* (n = 19), *E. coli* (n = 20), and *P. aeruginosa* (n = 16), from skin diseases of companion dogs (n = 206) and cats (n = 71) were obtained from the Animal and Plant Quarantine Agency (Gimcheon, Korea).

**Table 1:** *In vitro* antimicrobial activity of α-MG against *Staphylococcus* species and G (-) bacterial strains.

Bacteria	MIC (µg/ml)		MBC (µg/ml)
	Agar dilution	Broth microdilution	
*S. aureus* ATCC 29213	4	2	8
*S. carprae* KCTC 3583	2	2	4
*S. epidermidis* ATCC 12228	1	1	4
*S. felis* ATCC 49168	2	1	4
*S. intermedius* KCTC 3344	2	1	4
*S. pseudintermedius* ATCC 49051	2	1	2
*S. saprophyticus* KCTC 3345	2	1	2
*S. schleiferi* ATCC 43808	1	1	1
*E. coli* ATCC 25922	>64	>64	>64
*P. aeruginosa* ATCC 27853	>64	>64	>64

*MIC, minimum inhibitory concentration; MBC, minimum bactericidal concentration.

### Antimicrobial susceptibility testing

The broth microdilution method was used to determine the minimum inhibitory concentrations (MICs) and minimum bactericidal concentrations (MBCs) of α-MG. α-MG was dissolved in dimethyl sulfoxide (DMSO) (Sigma-Aldrich, St. Louis, MO, USA) to a concentration of 25 mg/ml and used as stock solution. The agar dilution method was also used to determine the MICs of α-MG. The antimicrobial activity testing of α-MG was performed by the guidelines of Clinical Laboratory Standards Institute (CLSI, 2020). Serial two-fold dilutions of α-MG were prepared in Mueller-Hinton agar or Mueller-Hinton broth (MHB; Difco, Detroit, MI, USA).

### Time-kill kinetics assay

Three bacterial strains (*S. felis* ATCC 49168, *S. pseudintermedius* ATCC 49051, and *S. schleiferi* ATCC 43808) were grown overnight in MHB at 37°C. Bacterial cultures were diluted to 1:100 in 10 ml MHB and 200 µl bacterial suspension was inoculated in each well in 96-well plates containing MHB with two-fold serially diluted α-MG (1/4× to 1× MIC). The plates were incubated at 37°C with shaking (150 rpm) for 18 h. The optical density at 600 nm (OD_600_) was measured every 1 h using a microplate reader (Molecular Devices, San Jose, CA, USA).

### Field emission-scanning electron microscopy

Three bacterial strains (*S. felis* ATCC 49168, *S. pseudintermedius* ATCC 49051, and *S. schleiferi* ATCC 43808) were grown overnight in lysogeny broth (LB) at 37°C. Each bacterial culture (10 µl) was inoculated in LB containing different α-MG concentrations (1/4×, 1/2×, 1×, 2×, and 4× MICs) in six-well plates with poly-L-lysine-coated cover glass (Sigma-Aldrich), and the plates were incubated at 37°C for 2 h. The cover glasses were removed from the plates and washed twice with phosphate-buffered saline (PBS). The samples were serially dehydrated with 20%, 50%, and 70% ethanol for 10 min, and 100% ethanol for 30 min. The samples were coated with platinum, and ultrastructural changes of bacteria were observed using FE-SEM (Hitachi SU-8220; Hitachi, Japan).

### Biotinylation of α-MG

α-MG was purchased from Combi-blocks (San Diego, CA, USA). For biotinylation of α-MG, biotin (82.4 mg, 0.33 mM) and hexafluorophosphate azabenzotriazole tetramethyl uranium (128.3 mg, 0.33 mM) in dimethylformamide (5 ml) and triethylamine (68.3 mg, 0.67 mM) were added in the stirred solution of α-MG (92.3 mg, 0.22 mM) at 0°C and stirred for 16 h at room temperature. The reaction mixture was diluted with CH_2_Cl_2_ (25 ml), and the solution was washed twice with distilled water (20 ml) and once with brine (20 ml). The sample solution was dried over anhydrous Na_2_SO_4_ and evaporated. The samples were purified by column chromatography (silica gel, 2%-5% methyl alcohol in CH_2_Cl_2_) to make the tile compound (100 mg, 51% yield) a pale-yellow solid. ^1^H nuclear magnetic resonance (400 MHz, CD_3_CN): δ_H_ 13.80 (s, 1H), 13.39 (s, 1H), 7.16 (s, 1H), 6.84 (s, 1H), 6.64 (s, 1H), 6.38 (s, 1H), 5.38 (d, *J* = 18.5 Hz, 3H), 5.23 (d, *J* = 1.4 Hz, 2H), 5.12 (t, *J* = 7.0 Hz, 1H), 4.53-4.38 (m, 2H), 4.35-4.20 (m, 2H), 4.16-4.02 (m, 3H), 3.77 (d, *J* = 7.9 Hz, 3H), 3.42-3.12 (m, 4H), 2.91 (ddd, *J* = 14.4, 7.2, 5.5 Hz, 2H), 2.68 (dt, *J* = 15.2, 7.1 Hz, 4H), 1.84-1.74 (m, 12H), 1.73-1.63 (m, 10H) ppm; mass spectrometry (MS)-electrospary ionization: *m/z* [M-H]^-^ calculated for C_34_H_39_N_2_O_8_S 635.24; found 635.26. Chemical structure of biotinylated α-MG was depicted in **Figure S1**.

### Protein pull-down assay

The protein pull-down assay was conducted using the Biotinylated Protein Interaction Pull-Down Kit (Thermo Fisher Scientific, Waltham, MA, USA). *S. pseudintermedius* ATCC 49051 was cultured overnight in LB at 37°C. After centrifugation of bacterial culture, the pellets were washed with PBS and suspended in buffer A [20 mM Tris-HCl (pH 7.5), 0.5 M NaCl]. Bacteria were lysed by sonication (Branson Sonifier 450; Branson Ultrasonics, Danbury, CT, USA). Biotinylated α-MG (20 mM) was added to streptavidin containing gel and incubated at 4°C for 2 h with gentle shaking. After removing the free biotinylated α-MG, the gel was blocked with a biotin solution for 5 min. Bacterial lysates were added to the gels and incubated at 4°C for 2 h with gentle shaking. The gel was washed twice with 0.25 M NaCl buffer, and bacterial proteins bound to biotinylated α-MG were obtained using an elution buffer.

### Liquid chromatography-MS

Protein samples were denatured by boiling at 95°C for 10 min and subjected to 10% sodium dodecyl sulfate-polyacrylamide gel electrophoresis (SDS-PAGE). Proteins were stained using a PowerStain Silver Stain Kit (Elpis, Daejeon, Korea). The specific protein bands were excised for subsequent gel digestion and analysis. Digested tryptic peptide samples were applied to 100 μm × 2 cm nanoViper trap columns (Thermo Fisher Scientific) to enrich the peptides and remove chemical contaminants. Concentrated tryptic peptides from the trap column were loaded to the 15 cm × 75 μm nanoViper analysis column (Thermo Fisher Scientific) at 300 nl/min flow rate. Peptides were eluted using a gradient from 0 to 65% acetonitrile for 80 min. All MS and MS/MS spectra were acquired in a data dependent mode using a Q Exactive Plus Hybrid Quadrupole-Orbitrap Mass Spectrometer (Thermo Fisher Scientific). Each full MS (*m/z* range of 300 - 2,000) scan was followed by three MS/MS scans of the most abundant precursor ions in MS spectra. MS/MS spectra were analyzed by MASCOT version 2.7 (Matrix Science, Boston, MA, USA) using protein sequences from the genome of *S. pseudintermedius* (NZ_CP065921.1) downloaded from the National Center for Biotechnology Information (NCBI; https://www.ncbi.nlm.nih.gov/).

### Production of recombinant major histocompatibility complex II analogous protein domain-containing proteins and mouse anti-MAP domain-containing protein polyclonal serum

The gene encoding the full-length MAP domain-containing protein (QIW05685.1 in *S. pseudintermedius* VTH737) was amplified by polymerase chain reaction (PCR) using the genomic DNA from *S. pseudintermedius* ATCC 49051 as a template. The specific primers (5´-GGG CGG CGG TGG TGG CGG CAT GAA AGC AAA AAA ATT ATT AGC TAC AGG-3´ and 5´-GTT CTT CTC CTT TGC GCC CTA TTA TTT CGC ACT TAC CAC TTC AAT-3´) were designed for ligation-independent cloning. After treating T4 DNA polymerase (New England Biolabs, Ipswich, MA, USA), the amplicons were inserted into pB3 plasmids derived from the pET21a plasmid (**Figure S2A**). The construction was confirmed by DNA sequencing analysis. The constructed plasmids were transformed into *E*. *coli* BL21 (DE3) cells and grown in LB to reach an OD_600_ of 0.4 - 0.5. The expression of recombinant proteins was induced with 0.2 mM isopropyl β-D-1-thiogalactopyranoside overnight at 18°C. The recombinant proteins were purified in a His-Trap HP column (GE Healthcare, Chicago, IL, USA). The recombinant proteins were purified by size exclusion chromatography (**Figure S2B**). The purified protein fractions were separated by 10% SDS-PAGE and stained with Coomassie blue G-250 (iNtRON Biotechnology, Seongnam, Korea) (**Figure S2C**). BALB/c mice (6-weeks old) were used to produce anti-MAP-domain containing protein polyclonal serum. The purified recombinant proteins (250 µg) were emulsified with the same volume of Freud’s complete adjuvant (FCA; Sigma-Aldrich) for the first injection of antigens and 50 µg proteins were emulsified with the same volume of Freud’s incomplete adjuvant (FIA; Sigma-Aldrich) for the boosting shot. Blood was obtained by retroorbital bleeding before immunization to prepare the pre-immune sera. On the first day, proteins emulsified with FCA were subcutaneously delivered to four sites of the mouse back. For boosting, proteins emulsified with FIA were injected similarly on the 21st and 42nd days. Whole blood was harvested on the 50th day.

### Bacterial killing in serum

*S. pseudintermedius* ATCC 49051 was cultured in trypticase soy agar (Difco) overnight at 37°C and resuspended in PBS. Bacteria [10^5^-10^6^ colony forming units (CFUs)/ml] were added to the antiserum immunized with recombinant MAP domain-containing protein prepared at a ratio of 1:500 and incubated at 37°C for 20 min. Different α-MG concentrations (1× to 4× MICs) were added to the tubes. The samples were collected at the indicated time points (0, 5, 10, and 20 min), and the CFU was determined. The experiments were performed in three independent experiments.

### Localization of MAP-domain containing protein

Whole cell lysates, cell wall-associated proteins, and membrane vesicles (MVs) were prepared from *S. pseudintermedius* ATCC 49051. To prepare whole cell lysates, bacteria were cultured in LB at 37°C for 16 h and bacterial cultures were centrifuged at 6,000 g at 4°C for 15 min. Bacteria were lysed by sonication. To prepare cell wall-associated proteins, bacteria were cultured in LB at 37°C for 16 h. After centrifugation, bacterial cells were placed on chilled ice for 5 min and resuspended in 5 ml TE buffer [50 mM Tris-HCl, 1 mM ethylenediaminetetraacetic acid (pH 8.0), and 1 mM phenylmethyl sulfonyl fluoride]. After centrifugation, bacterial cells were resuspended in 1.15 ml mutanolysin mixture [1 ml TES buffer (20% w/v sucrose in TE buffer), 100 µl lysozyme (100 mg/ml), and 50 µl mutanolysin (5,000 units/ml)] and incubated at 37°C for 2 h with shaking. The samples were centrifuged at 14,000 g for 5 min. MVs were prepared from bacterial culture supernatants as previously described (Jun et al., 2017). Bacteria were cultured in 1 L LB at 37°C with shaking to reach OD_600_ of 0.5 and MVs were purified. Purified MVs were checked for sterility on blood agar plates. Protein concentrations of the three samples were measured using the BCA assay (Thermo Fisher Scientific). Proteins (4 µg) were separated by 10% SDS-PAGE and electro transferred to a polyvinylidene difluoride membrane (Thermo Fisher Scientific). The membrane was blocked with a 5% skim milk solution in TBS-T buffer (20 mM Tris-HCl, 137 mM NaCl, and 0.01% Tween-20) for 2 h at room temperature. The membranes were treated with polyclonal antisera diluted with 1:1000 for 1 h and washed three times with TBS-T buffer. The membranes were incubated with secondary goat anti-mouse IgG-horseradish peroxidase-conjugated antibody diluted with 1:2000 (Ab Frontier, Seoul, Korea) for 1 h. The membrane was developed using SuperSignal™ West Pico Plus chemiluminescent substrates (Thermo Fisher Scientific).

### RNA extraction and transcriptome analysis

Transcriptome analysis of *S. pseudintermedius* treated with α-MG was performed as previously described with some modifications (Sivaranjani et al., 2019). *S. pseudintermedius* ATCC 49051 was grown to mid-log phase (~1.0 × 10^8^ CFU/ml) and treated with 0.7× MIC (0.7 µg/ml) of α-MG for 10 min. Bacteria were treated with 0.003% DMSO as a control. Total RNAs were prepared using a TRIzol reagent (Invitrogen, Carlsbad, CA, USA). The residual genomic DNA was digested by RNase-free DNase (Qiagen, Hilden, Germany). The structural integrity and quality of extracted RNAs were verified by an Agilent 2100 Bioanalyzer (Agilent Technologies, Santa Clara, CA USA). Two biological replicates were used to produce RNA samples for sequencing. To construct strand-specific libraries, rRNA was depleted using the Ribo-Zero rRNA Removal Kit (Illumina, San Diego, CA, USA). Libraries were constructed using a TruSeq Stranded Total RNA Sample Preparation Kit (Illumina) according to the manufacturer’s instructions. Briefly, rRNA-depleted RNAs were fragmented into short fragments (200 - 500 nt) using a Covaris S220 ultrasonicator (Woburn, MA, USA), and first-strand cDNA was synthesized from fragments using random hexamer primers and reverse transcriptase, followed by the synthesis of the second strand with dUTP substituted for dTTP. Ends of short fragments were processed by adding a single adenine after purification and connected with adapters. Finally, the second strand was degraded using uracil-N-glycosylase and PCR-amplified for eight cycles to construct strand-specific libraries. The constructed library was amplified with phi29 to make a DNA nanowall (DNB) with >300 copies of one molecule. DNBs were loaded into the patterned nanoarray, and paired-end 100 bases reads were generated via sequencing by combinatorial Probe-Anchor Synthesis using a DNBSEQ-T7 sequencing machine (BGI, Shenzhen, Guangdong, China). The quality of the raw reads (FASTQ) was evaluated using FastQC version 0.11.5 (https://www.bioinformatics.babraham.ac.uk/projects/fastqc/), and the dirty reads were removed before downstream analysis to decrease data noise. The reads were subjected to standard quality control criteria according to the following parameters: (1) trimming and cleaning reads that aligned to primers and/or adaptors, (2) reads with >50% of low-quality bases (quality value ≤5) in one read, and (3) reads with >10% unknown bases (N bases). After filtering, the remaining reads were called clean reads and stored in FASTQ format. All high-quality and clean reads were subjected to be mapped to the NCBI reference genome *S. pseudintermedius* ASM1612671v1 using HISAT to obtain the BAM file. BAM files were sorted and indexed with Samtools, and the number of reads matching each gene in *S. pseudintermedius* was counted with HTSeq-count (Anders et al., 2015; Kim et al., 2015). Read-count data were filtered (rowMax <1) and normalized using DESeq2 (version 1.26) and R packages (version 3.6.1) according to the protocol. Fold-change ratio and Wald test were performed between the control and test groups using DESeq2. Differentially expressed genes (DEGs) were considered when the fold-change ratio ≥2 or ≤0.5 and the Wald test *q <*0.05. Data visualization such as scatterplot, boxplot, volcano plot, and hit map was plotted using the R platform. The Kyoto Encyclopedia of Genes and Genomes (KEGG) pathway assignation was performed to determine the function of differentially regulated genes. Hypothetical proteins were analyzed for putative functions using protein BLAST to search for Pfam (protein families). Signal peptides and the location of cleavage sites in proteins were predicted using SignalP-5.0 (https://services.healthtech.dtu.dk/service.php?SignalP-5.0). Subcellular localization was predicted using PSORTb (https://psort.org/psortb). Three dimensional structure of protein was predicted using the AlphaFold program (http://alphafold.ebi.ac.uk).

### Quantitative real-time PCR

Total RNA from *S. pseudintermedius* ATCC 49051 was isolated as described above, and cDNA was synthesized using the M-MLV cDNA synthesis kit (Enzynomics, Daejeon, Korea). qPCR was performed using an ABI Step One Plus Real-Time System (Applied Biosystems, Waltham, MA, USA). TOPreal™ q-PCR 2X PreMix (SYBR Green with high ROX; Enzynomics) was used. Total RNA was also isolated from the dissected left ears of mice in *in vivo* experiments. The primers used to validate qPCR and the expression of cytokine genes are listed in **Supplementary Table S1**. The expression level was standardized against constitutively expressed 16S rRNA and glyceraldehyde 3-phosphate dehydrogenase expression levels and quantified using the 2^-△△Ct^ method. qPCR was performed in three independent experiments.

### Cell culture and cytotoxicity assay

Canine progenitor epidermal keratinocytes (CPEK) were purchased from CELLnTEC Advanced Cell System (Bern, Switzerland). CPEK cells were cultured in CnT-09 medium (CELLnTEC Advanced Cell System) at 37°C in a humidified 5% CO_2_ atmosphere. Cells were seeded in a 96-well plate at a density of 8.0 × 10^3^ cells/well. Cells were treated with different α-MG concentrations (0, 1, 4, 6, 10, 12, 14, and 16 µg/ml) for 24 h. Cell viability was measured using 3-(4,5-dimethylthiazol-2-yl)-2,5-diphenyltetrazolium bromide (MTT; Sigma-Aldrich). The optical density of each well was measured using a microplate reader at 550 nm. The experiments were performed in three independent experiments.

### α-MG in pluronic lecithin organogel

The aqueous phase was prepared by mixing pluronic F-127 (30% w/v) (Sigma-Aldrich) and potassium sorbate (0.2%) (Sigma-Aldrich) under magnetic stirring at 4°C. The oil phase containing 4 ml isopropyl palmitate (Sigma-Aldrich), lecithin (2% w/v) (DUKSAN Science, Seoul, Korea), and sorbic acid (0.2%) (Sigma-Aldrich) was homogenized under magnetic stirring at room temperature. α-MG (25 mg/ml) was added to the oil phase. To prepare PLO, the aqueous phase, oil phase, and polyethylene glycerol 600 (Sigma-Aldrich) were mixed at a ratio of 3.5:1:0.5 (v/v), respectively. All ingredients were homogenized using a luer lock syringe.

### Skin infection model

Female BALB/c mice (6 weeks old) were purchased from Hyochang Science (Daegu, Korea). Mice were housed in a flow chamber maintained at 22°C ± 2°C and relative humidity of 55% ± 5%. The tape-stripping infection model was used for skin infection of *S. pseudintermedius* in mice (Kugelberg et al., 2005). Two sets of four mice in each group were used in this study: one set of mice was used for qPCR and histological analyses, and the other set was used for CFU counting. *S. pseudintermedius* ATCC 49051 was cultured at 37°C for 18 h and diluted 1:100 in trypticase soy broth (Difco). Bacteria were grown to reach an OD_600_ of 0.5 and washed with PBS. After sterilizing the ears using 70% ethanol, the dorsal surface of both ears was stripped 30 times using the autoclave tape to disrupt the epidermal barrier. The bacterial suspension (1.0 × 10^9^ CFU/20 µl PBS) was inoculated on the stripped skin of both ears. For treatment group, α-MG (7.5 µg in 30 µl PLO) was applied four times with 6 h intervals to the infection sites 4 h after bacterial inoculation. Uninfected control was normal mice without the skin stripping, bacterial infection, or any treatment. Infected control mice (control group) were inoculated bacterial suspension on the stripped skin and then treated with DMSO in PLO (0.3 µl DMSO in 30 µl PLO) four times as the same intervals of treatment group. Animal experiments were performed in accordance with the regulations established by the Institutional Animal Care and Use Committee of Kyungpook National University.

### Histological analysis

To evaluate histological changes in the ears, mice were euthanized 6 h after the final treatment. The dissected right ears were fixed with 10% paraformaldehyde and embedded in a paraffin block. The paraffin-embedded ears were serially sectioned with 3-μm-thick slices and stained with hematoxylin and eosin (H&E).

### Statistical analysis

Statistical analyses were performed using GraphPad Prism 5.0 (San Diego, CA, USA). One-way analysis of variance with Dunnett’s *post-hoc* analysis and Student’s t-tests were performed to compare the control and experimental groups. Differences of *p <*0.05 were considered statistically significant.

## Results

### Antimicrobial activity of α-MG against *Staphylococcus* species and G (-) bacteria from companion animals *in vitro*

**Figure 1:**
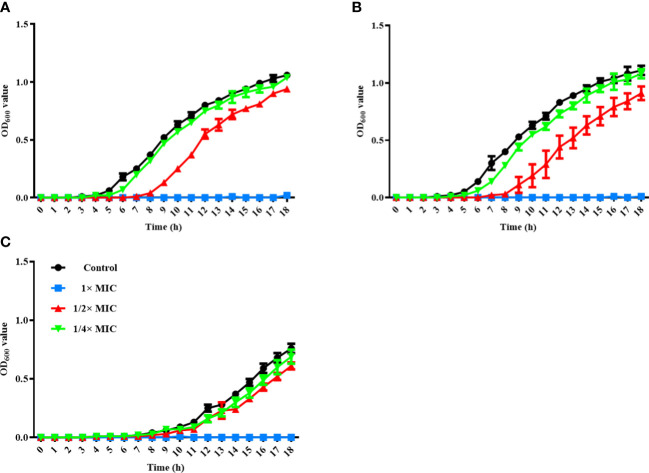
Time-kill kinetics assay for three *Staphylococcus* species. *S. felis* ATCC 49168 **(A)**, *S. pseudintermedius* ATCC 49051 **(B)**, and *S. schleiferi* ATCC 43808 **(C)** were grown in Mueller-Hinton broth with different α-MG concentrations (1/4×, 1/2×, and 1× MICs) for 18 h. OD_600_ was measured at the indicated time points. The data are presented as the mean ± SD of three independent experiments. The MIC values of α-MG against three *Staphylococcus* strains were 1 µg/ml using the broth microdilution method.

To assess the antimicrobial activity of α-MG against G (+) and G (-) bacterial species commonly isolated from skin diseases of companion animals, the MICs of α-MG against eight staphylococcal reference strains and two G (-) reference strains were determined using agar dilution and broth microdilution methods. The MICs of α-MG against staphylococcal strains ranged from 1 to 2 and 1 to 4 µg/ml in broth microdilution and agar dilution methods, respectively (**Table 1**). Five *Staphylococcus* species, including *S. aureus, S. felis, S. intermedius, S. pseudintermedius*, and *S. saprophyticus*, showed two-fold difference in MICs of α-MG between the broth microdilution and agar dilution methods, whereas three species, including *S. carprae, S. epidermidis*, and *S. schleiferi*, showed the same MIC values between the two methods. The MBCs of α-MG against *Staphylococcus* species were ranged from 1 to 8 µg/ml (**Table 1**). However, the MICs and MBCs of α-MG against *E. coli* ATCC 25922 and *P. aeruginosa* ATCC 27853 were >64 µg/ml. Next, the antimicrobial activity of α-MG against clinical isolates of *Staphylococcus* species and G (-) bacterial species derived from skin diseases of companion dogs and cats was determined using the agar dilution method. The MICs of α-MG against all clinical isolates of *Staphylococcus* species ranged from 1 to 16 µg/ml, whereas those against G (-) bacterial isolates were >64 µg/ml (**Table 2**). The MIC_50_ value of α-MG was the lowest in *S. schleiferi* isolates (4 µg/ml), whereas the MIC_90_ of α-MG was the highest in *S. aureus* isolates (16 µg/ml). Time-killing analysis was performed to understand the nature of the antimicrobial activity of α-MG against *S. felis*, *S. pseudintermedius* and *S. schleiferi*. The growth of the three staphylococcal strains was completely inhibited at the MIC of α-MG for 18 h (**Figure 1**). These results suggest that α-MG exhibits a strong antimicrobial activity against *Staphylococcus* species from companion animals *in vitro*, but not against G (-) bacteria.

**Table 2:** *In vitro* antimicrobial activity of α-MG against clinical isolates of *Staphylococcus* species and G (-) bacteria from skin lesions of companion dogs and cats.

Bacterial isolates (No.)	No of isolates with the following MIC (µg/ml)							MIC range (µg/ml)	MIC_50_ (µg/ml)	MIC_90_ (µg/ml)
	1	2	4	8	16	32	>64			
*S. aureus* (19)			1	12	6			4-16	8	16
*S. epidermidis* (27)	1		5	20	1			1-16	8	8
*S. felis* (51)		1	9	41				2-8	8	8
*S. pseudintermedius* (80)	1		22	57				1-8	8	8
*S. schleiferi* (64)	1	3	48	12				1-8	4	8
*E. coli* (20)							20	>64	>64	>64
*P. aeruginosa* (16)							16	>64	>64	>64

*MIC, minimum inhibitory concentration; MIC_50_, MIC of 50% of the isolates; MIC_90_, MIC of 90% of the isolates.

### Morphological changes of *Staphylococcus* species treated with α-MG

**Figure 2:**
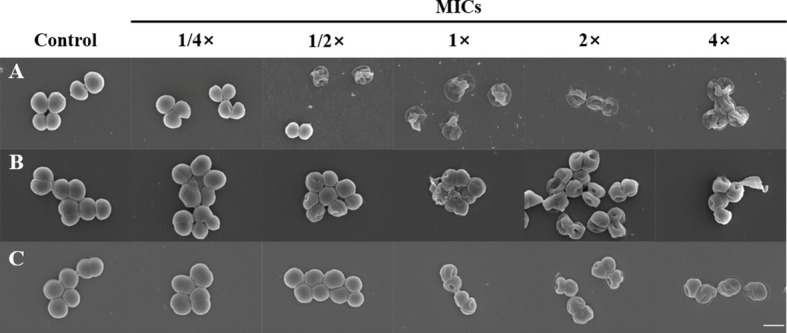
Morphological changes of bacteria treated with α-MG. *S. felis* ATCC 49168 **(A)**, *S. pseudintermedius* ATCC 49051 **(B)**, and *S. schleiferi* ATCC 43808 **(C)** were treated with 1/4×, 1/2×, 1×, 2×, and 4× MICs of α-MG for 2 h, and bacterial morphology was analyzed using FE-SEM. The control bacteria were treated with 0.16% DMSO. The MIC values α-MG against three *Staphylococcus* strains were 1 µg/ml using the broth microdilution method. Magnification, ×20,000. Scale bar, 1 μm.

To determine whether α-MG induced the morphological changes of bacteria, three staphylococcal strains were treated with different α-MG concentrations (1/4× to 4× MIC) for 2 h, and bacterial morphology was assessed using FE-SEM. No morphological change was observed in bacteria treated with 1/4× MIC of α-MG compared to bacteria treated with DMSO, a solvent of α-MG (**Figure 2**). However, morphological changes were observed in *S. felis* and *S. pseudintermedius* treated with ≥1/2× MICs of α-MG and *S. schleiferi* treated with ≥1× MICs of α-MG. Control bacteria showed an intact and smooth surface and a spherical shape, whereas staphylococcal strains treated with ≥1× MICs of α-MG showed significant morphological changes such as deep surface craters, cell disruption, and cellular debris.

### Specific binding of α-MG to MAP domain-containing protein in the cytoplasmic membrane of *S. pseudintermedius*

**Figure 3:**
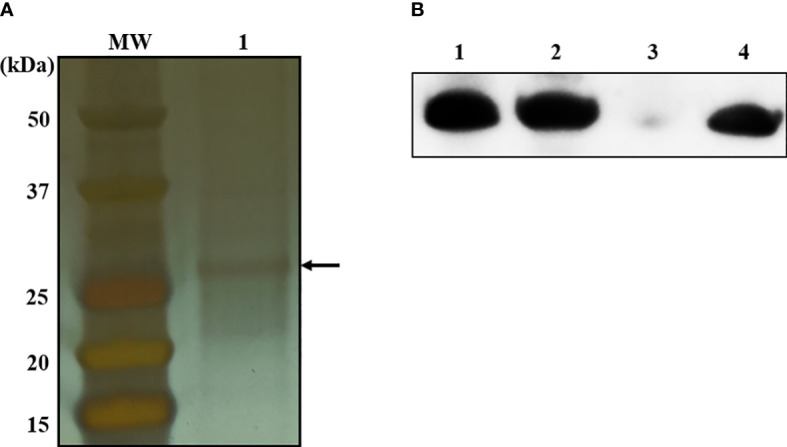
Bacterial proteins interacting with α-MG labeled with one biotin molecule at C-3 or C-6 and subcellular localization of MAP domain-containing protein. **(A)** Whole cell lysates of *S. pseudintermedius* ATCC 49051 were added to streptavidin gels bound to α-MG labeled with one biotin molecule. Bacterial proteins bound to biotinylated α-MG were observed in silver-stained SDS-PAGE gel. Arrow indicates bacterial proteins bound to biotinylated α-MG. MW, molecular weight size marker; 1, bacterial proteins bound to biotinylated α-MG. **(B)** Subcellular localization of MAP domain-containing protein using western blot analysis. Bacterial proteins of *S. pseudintermedius* ATCC 49051 were separated by 10% SDS-PAGE and western blot was performed using the mouse anti-MAP domain-containing protein polyclonal serum. Lanes 1, whole cell lysates; 2, cell wall-associated proteins; 3, membrane vesicles; 4, recombinant MAP domain-containing proteins.

To identify bacterial molecules interacting with α-MG, α-MG was labeled with biotin molecules at the hydroxyl group (-OH) of C-3 and/or C-6. The antimicrobial activity of biotinylated α-MG was first determined against *S. felis* ATCC 49168, *S. pseudintermedius* ATCC 49051, and *S. schleiferi* ATCC 43808. The MICs of α-MG labeled with one biotin molecule at either C-3 or C-6 increased from 1 to 32 µg/ml in the three staphylococcal strains using the broth microdilution method. Moreover, the MICs of α-MG labeled with two biotin molecules at both sites increased to 32 µg/ml in *S. felis* ATCC 49168, 64 µg/ml in *S. schleiferi* ATCC 43808, and 128 µg/ml in *S. pseudintermedius* ATCC 49051. These results suggest that hydroxyl groups at C-3 and C-6 are essential for the antimicrobial activity of α-MG against *Staphylococcus* species. Next, a protein pull-down assay was performed using biotinylated α-MG. Streptavidin gels were used in this assay due to the high affinity of biotin to streptavidin. Whole cell lysates of *S. pseudintermedius* ATCC 49051 were incubated with α-MG labeled with one biotin molecule bound to streptavidin gels. Bacterial proteins with ~27 kDa were identified to interact with biotinylated α-MG in SDS-PAGE gels (**Figure 3A**). However, no protein band was observed when bacterial lysates were incubated with α-MG labeled with two biotin molecules at C-3 and C-6 (**Figure S3**). Four bacterial proteins were identified (**Table 3**). The MAP domain-containing protein was the most abundant of the four proteins and was predicted to be located in the cytoplasmic membrane using the PSORTb program. To determine the exact localization of MAP domain-containing protein in bacteria, whole cell lysates, cell wall-associated proteins, and MVs were purified from *S. pseudintermedius* ATCC 49051, and western blot analysis was performed using the mouse anti-MAP domain-containing protein polyclonal serum. This revealed that the MAP domain-containing protein was located in the cell wall, but this protein was merely secreted by MVs (**Figure 3B**). These results suggest that α-MG interacts with MAP domain-containing protein located in the cytoplasmic membrane of *S. pseudintermedius* via hydroxyl groups at C-3 and C-6.

**Table 3:** Identification of bacterial proteins bound to biotinylated α-MG using LC-MS.

Gene accession No.^a^	Description of the identified protein	MW (Da)	pI	Signal peptide^b^	Subcellular localization^c^	emPAI^d^
WP_105963101.1	helix-turn-helix transcriptional regulator [*Staphylococcus*]	25,492	4.89	No	Cytoplasm	0.19
WP_014613648.1	MAP domain-containing protein [*S. pseudintermedius*]	27,424	9.86	Yes	Cytoplasmic membrane	4.36
WP_110159498.1	SAR2788 family putative toxin [*S. pseudintermedius*]	27,559	9.48	Yes	Extracellular	0.16
WP_014612681.1	response regulator transcription factor [*Staphylococcus*]	27,322	5.17	No	Cytoplasm	0.18

a NCBI reference sequence (https://www.ncbi.nlm.nih.gov/).

b Prediction of signal peptide and cleavage sites by SignalP 3.0 (https://services.healthtech.dtu.dk/service.php?SignalP-5.0).

c Prediction of cellular location by PSORTb v3.0 (https://psort.org/psortb).

d Exponentially modified protein abundance index.

### Antimicrobial activity of α-MG by the interaction with MAP domain-containing protein

**Figure 4:**
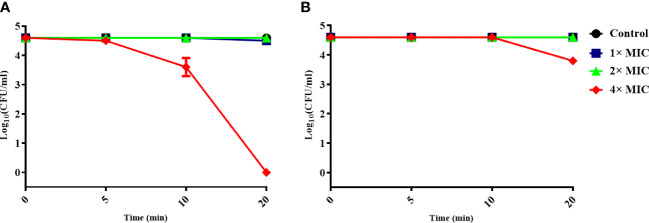
Inhibition of the antimicrobial activity of α-MG by binding anti-MAP domain-containing protein polyclonal serum to bacteria. *S. pseudintermedius* ATCC 49051 was pretreated with mouse pre-immune serum **(A)** or anti-MAP domain-containing protein polyclonal serum **(B)** at a dilution ratio of 1:500 for 20 min, and then bacteria were treated with different α-MG concentrations for 20 min. The samples were washed with PBS, and CFU was calculated. The data are presented as mean ± SD of three independent experiments.

To determine whether the antimicrobial activity of α-MG was mediated by the interaction with the MAP domain-containing protein, *S. pseudintermedius* ATCC 49051 was pretreated with mouse anti-MAP domain-containing protein polyclonal serum for 20 min and incubated with different α-MG concentrations (1×, 2×, and 4× MICs) for 20 min. As a control, bacteria were pretreated with mouse pre-immune serum and treated with α-MG. No significant difference in the CFU of bacteria treated with 1× and 2× MICs of α-MG was observed between the pretreatment of pre-immune serum and anti-MAP domain-containing protein polyclonal serum (**Figure 4**). α-MG with 4× MIC completely killed bacteria pretreated with pre-immune serum within 20 min, but the same α-MG concentration reduced the CFU <10-fold in bacteria pretreated with anti-MAP domain-containing protein polyclonal serum. These results suggest that the antimicrobial activity of α-MG against *S. pseudintermedius* is directly mediated by the interaction with MAP domain-containing proteins.

### Gene expression of *S. pseudintermedius* ATCC 49051 treated with sub-MIC of α-MG

**Figure 5:**
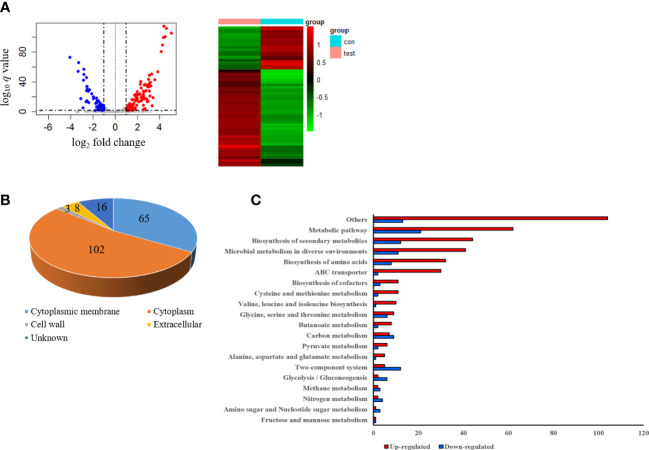
Differentially expressed genes (DEGs) in *S. pseudintermedius* ATCC 49051 exposed to sublethal concentration of α-MG. Bacteria were treated with 0.7× MIC of α-MG for 10 min, and total RNA was isolated. As a control, bacteria were treated with DMSO. **(A)** Volcano plot and DEGs. **(B)** Prediction of subcellular localization of proteins encoded by DEGs. **(C)** Upregulated and downregulated genes classified by the KEGG pathway.

To investigate the gene expression of *S. pseudintermedius* exposed to sub-MIC of α-MG, *S. pseudintermedius* ATCC 49051 was treated with 0.7× MIC of α-MG for 10 min, and DEGs were analyzed. As a control, bacteria were treated with DMSO. A total of 2,253 genes were identified under control and experimental conditions, corresponding to 91.5% (2,463 genes) of *S. pseudintermedius* genes. Of the 2,253 genes identified, 194 genes, which showed a fold change ≥2 or ≤0.5 and Wald test *q <*0.05, were identified as DEGs using DEseq2 analysis (**Figure 5A**). Upregulated and downregulated genes were 135 and 59, respectively. DEGs of *S. pseudintermedius* ATCC 49051 exposed to sub-MIC of α-MG are listed in **Supplementary Table S2**. qPCR was performed to verify the expression of DEGs by transcriptome analysis. The four upregulated and downregulated genes showed the same expression pattern observed in the transcriptome analysis (**Figure S4**). Cytoplasmic proteins were the most abundant (52.6%), followed by cytoplasmic membrane proteins (33.5%) (**Figure 5B**). Based on KEGG pathways, DEGs involved with metabolic pathways were abundant, followed by biosynthesis of secondary metabolites (**Figure 5C**). However, many DEGs were found to encode hypothetical proteins. These results suggest that the sublethal concentration of α-MG regulates the expression of many genes, especially in metabolic pathway, in *S. pseudintermedius*.

### Host cell toxicity of α-MG *in vitro*

**Figure 6:**
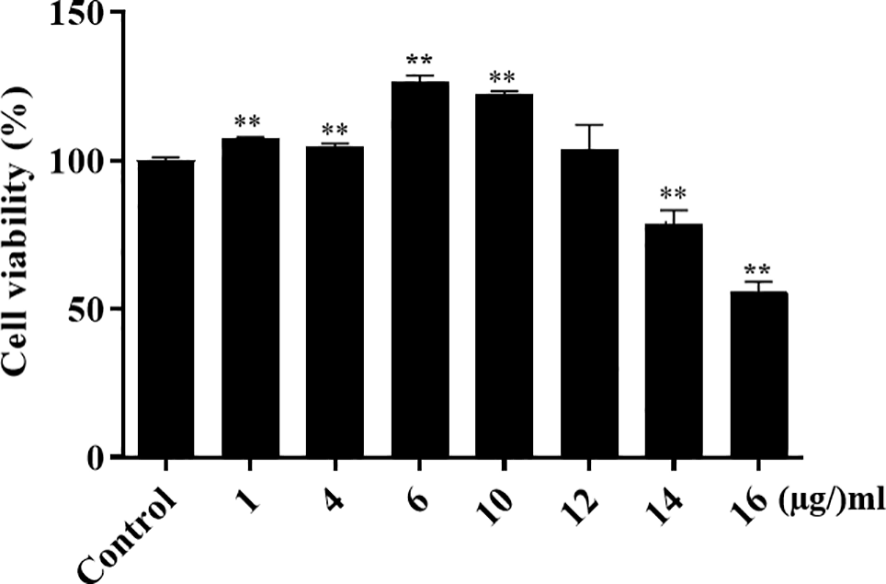
Viability of canine progenitor epidermal keratinocytes treated with α-MG. Cells were treated with different α-MG concentrations (1 - 16 µg/ml) for 24 h, and MTT assays were performed. Control cells were treated with 0.13% DMSO. The data are presented as mean ± SD of three independent experiments. **p < 0.01 compared to control cells.

To determine whether α-MG could induce host cell cytotoxicity, CPEKs were treated with different α-MG concentrations (1-16 µg/ml) for 24 h, and cell viability was determined using the MTT assay. As a control, CPEKs were treated with DMSO. No cytotoxicity was observed at ≤12 µg/ml α-MG, but the cytotoxicity was observed at ≥14 µg/ml α-MG (**Figure 6**).

### Antimicrobial and anti-inflammatory activities of α-MG in skin lesions caused by *S. pseudintermedius in vivo*

**Figure 7:**
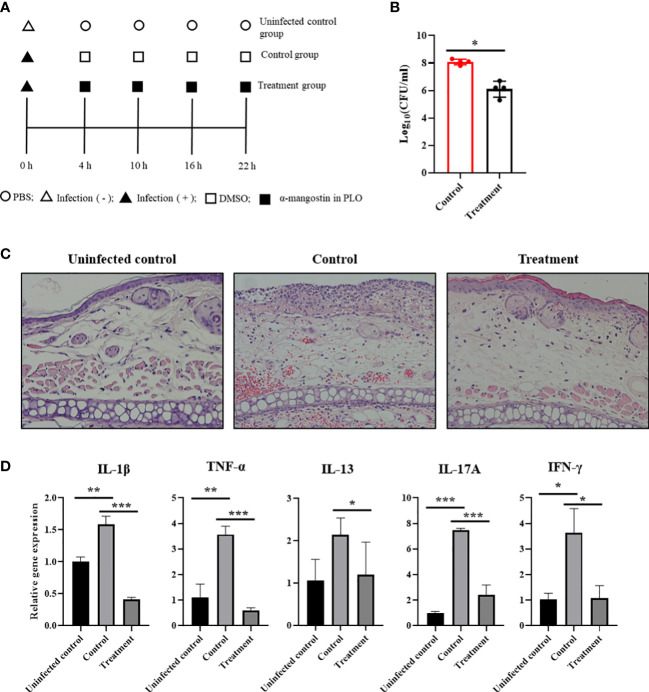
Antimicrobial and anti-inflammatory activities of α-MG in skin lesions induced by *S. pseudintermedius* in a murine model. **(A)** Experimental schedule for the application of α-MG. Mice were infected with 1.0 × 10^9^ CFUs/20 µl *S. pseudintermedius* ATCC 49051 and treated with α-MG in PLO. Infected control mice (control) were treated with DMSO in PLO. **(B)** Antimicrobial activity of α-MG in the tape-stripping infection model. The data are presented as mean of CFUs ± SD of four mice. *p < 0.05 compared to the control group. **(C)** Histopathological assessment of mouse ear. The ear section was stained with H&E. Magnification, ×200. **(D)** Expression of cytokine genes using qPCR. The data are presented as mean ± SD of four mice. *p < 0.05, **p < 0.01, and ***p < 0.001 compared to uninfected or infected control group.

To determine the therapeutic potentials of α-MG in bacterial skin infections *in vivo*, *S. pseudintermedius* ATCC 49051 was inoculated on the dorsal side of ears in mice, and α-MG in PLO was applied four times at 6 h intervals (**Figure 7A**). PLO was used for the topical drug delivery vehicle of α-MG. CFU, histological changes, and expression of cytokine genes in skin lesions were assessed 6 h after the final treatment. α-MG in PLO reduced the bacterial number by >99% compared to control mice treated with DMSO in PLO (**Figure 7B**). Infection of *S. pseudintermedius* ATCC 49051 induced the loss of the epidermal barrier and infiltration of cells in skin lesions of the infected control group (**Figure 7C**). α-MG in PLO reduced the number of infiltrating cells and partially restored the epidermal structure in skin lesions. To determine the anti-inflammatory activity of α-MG in PLO, qPCR was performed to measure the expression of pro-inflammatory (IL-1β and TNF-α), Th1 (IFN-γ), Th2 (IL-13), and Th17 (IL-17A) cytokine genes. *S. pseudintermedius* infection (control group) significantly induced the expression of all tested cytokine genes, but α-MG in PLO significantly inhibited the expression of all tested cytokine genes like uninfected control group (**Figure 7D**). These results suggest that α-MG in PLO exhibits antimicrobial and anti-inflammatory activities in skin lesions caused by *S. pseudintermedius in vivo*.

## Discussion

The antimicrobial activity of α-MG against G (+) bacterial pathogens, including *S. aureus*, *S. epidermidis, Enterococcus faecalis*, and *Streptococcus gordonii* from human clinical specimens, has already been demonstrated (Sakagami et al., 2005; Sivaranjani et al., 2017; Felix et al., 2022; Leelapornpisid, 2022). However, the antimicrobial activity of α-MG against *Staphylococcus* species originating from companion animals has not been characterized. The MICs of α-MG against reference strains of *Staphylococcus* species and G (-) bacterial species were first determined using the broth microdilution and agar dilution methods. There was no or 2-fold difference in MICs of α-MG between the two methods. Time-kill kinetics showed the different growth inhibition pattern of *S. schleiferi* treated 1/2 MIC of α-MG compared to that of *S. pseudintermedius* and *S. felis* with 1/2 MIC of α-MG. This is partly due to slow growth of *S. schleiferi in vitro* culture. The agar dilution method was applied to determine the MICs of α-MG against 241 isolates of five different *Staphylococcus* species from skin diseases of companion dogs and cats. The distribution of MICs of α-MG against clinical isolates of all tested *Staphylococcus* species represents a strong antimicrobial activity of α-MG *in vitro*. However, α-MG showed high MICs (>64 μg/ml) against clinical isolates of *E. coli* and *P. aeruginosa* from companion animals. These results suggest that α-MG exhibits antimicrobial activity against *Staphylococcus* species from companion animals as well as G (+) bacterial pathogens from humans (Sakagami et al., 2005; Sivaranjani et al., 2017; Felix et al., 2022; Leelapornpisid, 2022).

To understand the action mechanisms of α-MG against *Staphylococcus* species, FE-SEM was used to determine the morphological changes of bacteria treated with α-MG. Interestingly, ultrastructural alterations of bacteria treated with α-MG such as surface craters, surface indentation, cell disruption, and cellular debris were comparable to the results from *S. aureus* treated with antimicrobial peptides (Wu and Hancock, 1999; Tan et al., 2019; Deshmukh et al., 2021) or membrane acting antimicrobials such as daptomycin (Silverman et al., 2003), suggesting that α-MG targets the cytoplasmic membrane of *Staphylococcus* species. α-MG causes considerable damage to membrane integrity, whereas membrane acting antimicrobials produce ion channels or transmembrane pores (Wu and Hancock, 1999; Silverman et al., 2003; Cotroneo et al., 2008). Koh et al. (2013) also demonstrated that α-MG directly interacts with the cytoplasmic membrane of *S. aureus *via non-electrostatic interactions, causing bacterial lysis. However, the specific targets of α-MG in the cytoplasmic membrane have not been determined. To identify the specific interacting molecules of α-MG in the cytoplasmic membrane of *S. pseudintermedius*, hydroxyl groups of α-MG at C-3 and C-6 were substituted with biotin molecules. The hydroxyl groups of α-MG at C-3 and C-6 were selected because acetylation at C-3 and C-6 significantly changed the pharmacological activities of α-MG such as antioxidant and anticancer properties in human cells (Tran et al., 2021). Methylation at C-3 and C-6 in α-MG and γ-mangostin also reduced antimicrobial activity against G (+) bacteria (Tran et al., 2021). However, α-MG maintained antimicrobial activity when an appropriate cationic moiety or aliphatic amine was attached to the hydrophobic core and lipophilic chains instead of hydroxyl groups (Zou et al., 2013; Koh et al., 2015). The antimicrobial activity of biotinylated α-MG was first tested against three *Staphylococcus* species. Biotinylation α-MG at C-3 and C-6 showed lower antimicrobial activity against *Staphylococcus* species than α-MG. The MICs of α-MG with two biotin molecules were higher than those of α-MG with one biotin. Moreover, α-MG with one biotin molecule interacted with bacterial proteins, whereas no bacterial proteins were found to interact with α-MG with two biotin molecules. These results suggest that α-MG interacts with bacterial proteins via hydroxyl groups at C-3 and C-6.

In this study, MAP domain-containing protein was identified to interact with α-MG. MAP shows that six repeated domains of 110 residues containing a subdomain of 31 residues are highly homologous to the N-terminal *β*-chain of many MHC class II molecules (Jonsson et al., 1995; Kreikemeyer et al., 2002; Haggar et al., 2003). Mice infected with the *map*-negative mutant strain exhibit a reduced kidney abscess formation compared to mice infected with the wild-type *S. aureus* strain (Lee et al., 2002). MAP may attenuate cellular immunity and potentiate *S. aureus* survival by modulating host immunity. However, the MAP domain-containing protein identified in *Staphylococcus* species has not been characterized yet. The MAP domain-containing protein was predicted to carry a signal peptide (1-30 amino acids) and two MAP domain regions (37-147 and 148-247 amino acids) using the AlphaFold program (**Figure S5**). The sequence homology of amino acids was searched to determine whether MAP domain-containing protein was identified only in G (+) bacteria because α-MG exhibited antimicrobial activity against only G (+) bacteria, but not G (-) bacteria. The amino acid sequence of the MAP domain-containing protein identified in *S. pseudintermedius* ATCC 49051 was similar to 61%, 33%, 33%, and 30% in *Staphylococcus*, *Bacillus*, *Streptomyces*, and *Clostridium* species, respectively, using the UniProt BLAST program (**Figure S6**). However, this protein did not show any similarity to proteins in G (-) bacteria. These results may partly explain the antimicrobial activity of α-MG against G (+) bacteria, but not G (-) bacteria. Blocking the MAP domain-containing protein in the cytoplasmic membrane of *S. pseudintermedius* by anti-MAP domain-containing protein polyclonal serum significantly reduced the antimicrobial activity of α-MG. For the first time, this study demonstrated that α-MG specifically interacts with MAP domain-containing protein in the cytoplasmic membrane of *S. pseudintermedius*, resulting in cell lysis.

The subinhibitory concentrations of α-MG change the expression of many genes in *S. epidermidis* (Sivaranjani et al., 2019). Transcriptomic and proteomic analyses of *S. epidermidis* treated with sub-MIC (0.7×) of α-MG show downregulation of genes conferring cytoplasmic membrane integrity, cell division, teichoic acid biosynthesis, fatty acid biosynthesis, biofilm formation, and DNA replication and repair machinery, whereas sub-MIC of α-MG upregulates the genes associated with oxidative and cellular stress responses. In this study, the sub-MIC (0.7×) of α-MG modulated the expression of 194 genes in *S. pseudintermedius*, especially genes associated with metabolic pathways. α-MG upregulated genes encoding fatty acid biosynthesis, fatty acid degradation, fatty acid metabolism, pyrimidine (*carB, dcd,pyrD, pyrE, pyrF*, and *pyrK*), nucleotide metabolism (*dcd*), and CoA biosynthesis (*ilvB, ilvD, ilvE*, and *ilvN*), essential for bacterial survival. α-MG downregulated genes encoding pentose phosphate (fructose bisphosphate aldolase, *pfkA*), peptidoglycan biosynthesis (*sgtB* and *spsN*), teichoic acid biosynthesis (*dltX*), and citrate cycle (2-oxoacid: acceptor oxidoreductase subunit α). In addition, α-MG upregulated genes associated with siderophores (*FhuD, sbnA, sbnB, sbnC, sbnE*, and *sbnF*), but downregulated virulence determinants, including exotoxin (SpEX), β-channel forming cytolysin (leukocidin), leukocidin/hemolysin toxin family protein, and coagulase (Cheng et al., 2010; Abouelkhair et al., 2018 and Abouelkhair et al., 2019). These results suggest that sublethal concentrations of α-MG can modulate genes associated with the metabolic pathway, cell wall biosynthesis, and virulence factors in *S. pseudintermedius*, although the cytoplasmic membrane is a primary target of α-MG. It should be determined whether the transcriptional regulation of α-MG is a direct effect induced by the interaction with regulatory molecules in the cytoplasm or an indirect effect induced by the interaction with bacterial molecules in the cytoplasmic membrane.

The tape-stripping infection model was used to determine the therapeutic potential of α-MG *in vivo*, because *Staphylococcus* species was commonly associated with superficial and surface pyoderma in companion animals (Empinotti et al., 2012). By inoculation of *S. pseudintermedius*, high CFUs were maintained and infiltrating cells were remarkably observed in skin lesions. A relatively low dose of α-MG (7.5 µg in PLO) was applied in skin lesions because host cell cytotoxicity was induced in CPEKs treated with ≥14 µg/ml α-MG *in vitro*, and MICs of α-MG against all clinical isolates of *S. pseudintermedius* were ≤8 µg/ml. α-MG induced cellular proliferation of CPEKs treated with 1-10 µg/ml α-MG. α-MG has an antitumor activity against various types of cancer cells, but α-MG induces proliferation of non-tumorigenic human keratinocytes (HaCaT cells) after ultraviolet B (UVB) radiation (Im et al., 2017). α-MG may modulate innate immunity by the proliferation of keratinocytes induced by *S. pseudintermedius* infection like UVB radiation. α-MG in PLO significantly reduced bacterial number in skin lesions. In addition, α-MG in PLO significantly decreased the expression of cytokine genes associated with inflammation and adaptive immunity. Partial restoration of the epidermal structure and disappearance of infiltrated cells were observed in skin lesions treated with α-MG in PLO. These results suggest that α-MG in PLO exerts a synergistic effect on *S. pseudintermedius*-induced skin lesions via antimicrobial and anti-inflammatory activities in a murine model. Xanthones modulate inflammatory responses and the expression of inflammatory cytokines (Aye et al., 2020; Gunter et al., 2020). Oral administration of mangosteen rind extract inhibits the expression of pro-inflammatory cytokine and atopic dermatitis-associated genes and restores the epidermal barrier in an atopic dermatitis murine model (Higuchi et al., 2013). In addition, xanthones, including α-MG, exhibit anti-acne activity induced by *Cutibacterium acnes* (Chomnawang et al., 2007; Xu et al., 2018). However, complete clearance of bacteria was not observed in skin lesions, although α-MG concentrations exceeded the MIC level against *S. pseudintermedius* ATCC 49051. The lower antimicrobial activity of α-MG against *S. pseudintermedius in vivo* than its antimicrobial activity *in vitro* may be due to insolubility or low binding of α-MG to bacterial surface in skin lesions.

In conclusion, α-MG exhibited antimicrobial activity against clinical isolates of *Staphylococcus* species from companion animals *in vitro*. α-MG specifically interacted with MAP domain-containing protein in the cytoplasmic membrane of *S. pseudintermedius* via hydroxyl groups at C-3 and C-6. In addition, sublethal concentrations of α-MG inhibited the expression of genes conferring virulence factors in *S. pseudintermedius*. α-MG in PLO was an effective treatment modality in skin lesions caused by *S. pseudintermedius *via antimicrobial and anti-inflammatory activities. The development of new α-MG therapeutics is a novel approach to treating *Staphylococcus*-induced skin infections in companion animals, reducing the use of conventional antimicrobial agents.

## Data availability statement

The data presented in the study are deposited in the NCBI repository, accession number PRJNA963078.

## Ethics statement

Animal experiments were performed in accordance with the regulations established by the Institutional Animal Care and Use Committee of Kyungpook National University.

## Author contributions

SYP, JHL, and JCL designed the experiments. SYP, SYK, NK, and SYK carried out the experiments. JCL directed research and provided insightful discussions. All authors contributed to the analysis of the data, revised the manuscript, and approved its final version.

## References

[B1] Abouelkhair M. A. Bemis D. A. Giannone R. J. Frank L. A. Kania S. A. (2018). Characterization of a leukocidin identified in *Staphylococcus pseudintermedius* . PloS One 13, e0204450. doi: 10.1371/journal.pone.0204450 30261001 PMC6160070

[B2] Abouelkhair M. A. Bemis D. A. Giannone R. J. Frank L. A. Kania S. A. (2019). Identification, cloning and characterization of SpEX exotoxin produced by *Staphylococcus pseudintermedius* . PloS One 14, e0220301. doi: 10.1371/journal.pone.0220301 31356636 PMC6663030

[B3] Akao Y. Nakagawa Y. Nozawa Y. (2008). Anti-cancer effects of xanthones from pericarps of mangosteen. Int. J. Mol. Sci. 9, 355–370. doi: 10.3390/ijms9030355 19325754 PMC2635669

[B4] Anders S. Pyl P. T. Huber W. (2015). HTSeq-a Python framework to work with high-throughput sequencing data. Bioinformatics 31, 166–169. doi: 10.1093/bioinformatics/btu638 25260700 PMC4287950

[B5] Animal and Plant Quarantine Agency (2020). National surveillence of antimicrobial use and antimicrobial resistance monitoring (Gimcheon, Korea: Animal and Plant Quarantine Agency. Mama Communication Publisher).

[B6] Aye A. Song Y. J. Jeon Y. D. Jin J. S. (2020). Xanthone suppresses allergic contact dermatitis *in vitro* and *in vivo* . Int. Immunopharmacol. 78, 106061. doi: 10.1016/j.intimp.2019.106061 31821937

[B7] Bannoehr J. Guardabassi L. (2012). *Staphylococcus pseudintermedius* in the dog: taxonomy, diagnostics, ecology, epidemiology and pathogenicity. Vet. Dermatol. 23, 253–266. doi: 10.1111/j.1365-3164.2012.01046.x 22515504

[B8] Bennani H. Mateus A. Mays N. Eastmure E. Stark K. D. C. Hasler B. (2020). Overview of evidence of antimicrobial use and antimicrobial resistance in the food chain. Antibiotics 9, 49. doi: 10.3390/antibiotics9020049 32013023 PMC7168130

[B9] Cavana P. Robino P. Stella M. C. Bellato A. Crosaz O. Fiora S. R.. (2023). Staphylococci isolated from cats in Italy with superficial pyoderma and allergic dermatitis: characterisation of isolates and their resistance to antimicrobials. Vet. Dermatol. 34, 14–21. doi: 10.1111/vde.13127 36221849

[B10] Cheng A. G. McAdow M. Kim H. K. Bae T. Missiakas D. M. Schneewind O. (2010). Contribution of coagulases towards *Staphylococcus aureus* disease and protective immunity. PloS Pathog. 6, e1001036. doi: 10.1371/journal.ppat.1001036 20700445 PMC2916881

[B11] Chomnawang M. T. Surassmo S. Nukoolkarn V. S. Gritsanapan W. (2007). Effect of *Garcinia mangostana* on inflammation caused by *Propionibacterium acnes* . Fitoterapia 78, 401–408. doi: 10.1016/j.fitote.2007.02.019 17644272

[B12] Clinical and Laboratory Standards Institute (2020). Performance standards for antimicrobial susceptibility testing. 30th Edition (Wayne, PA, USA: CLSI).

[B13] Cotroneo N. Harris R. Perlmutter N. Beveridge T. Silverman J. A. (2008). Daptomycin exerts bactericidal activity without lysis of *Staphylococcus aureus* . Antimicrob. Agents Chemother. 52, 2223–2225. doi: 10.1128/AAC.01410-07 18378708 PMC2415783

[B14] Deshmukh R. Chalasani A. G. Chattopadhyay D. Roy U. (2021). Ultrastructural changes in methicillin-resistant *Staphylococcus aureus* (MRSA) induced by a novel cyclic peptide ASP-1 from *Bacillus subtilis*: a scanning electron microscopy (SEM) study. Rev. Argent Microbiol. 53, 281–286. doi: 10.1016/j.ram.2020.11.006 33608109

[B15] Devriese L. A. Hermans K. Baele M. Haesebrouck F. (2009). *Staphylococcus pseudintermedius* versus *Staphylococcus intermedius* . Vet. Microbiol. 133, 206–207. doi: 10.1016/j.vetmic.2008.06.002 18760884

[B16] Empinotti J. C. Uyeda H. Ruaro R. T. Galhardo A. P. Bonatto D. C. (2012). Pyodermitis. Bras. Dermatol. 87, 277–284. doi: 10.1590/S0365-05962012000200013 22570033

[B17] Felix L. Mishra B. Khader R. Ganesan N. Mylonakis E. (2022). *In vitro* and *In vivo* bactericidal and antibiofilm efficacy of alpha-mangostin against *Staphylococcus aureus* persister cells. Front. Cell Infect. Microbiol. 12, 898794. doi: 10.3389/fcimb.2022.898794 35937701 PMC9353584

[B18] Gunter N. V. Teh S. S. Lim Y. M. Mah S. H. (2020). Natural xanthones and skin inflammatory diseases: multitargeting mechanisms of action and potential application. Front. Pharmacol. 11, 594202. doi: 10.3389/fphar.2020.594202 33424605 PMC7793909

[B19] Haggar A. Hussain M. Lonnies H. Herrmann M. Norrby-Teglund A. Flock J. I. (2003). Extracellular adherence protein from *Staphylococcus aureus* enhances internalization into eukaryotic cells. Infect. Immun. 71, 2310–2317. doi: 10.1128/IAI.71.5.2310-2317.2003 12704099 PMC153217

[B20] Higuchi H. Tanaka A. Nishikawa S. Oida K. Matsuda A. Jung K.. (2013). Suppressive effect of mangosteen rind extract on the spontaneous development of atopic dermatitis in NC/Tnd mice. J. Dermatol. 40, 786–796. doi: 10.1111/1346-8138.12250 24033377

[B21] Ibekwe A. M. Bhattacharjee A. S. Phan D. Ashworth D. Schmidt M. P. Murinda S. E.. (2023). Potential reservoirs of antimicrobial resistance in livestock waste and treated wastewater that can be disseminated to agricultural land. Sci. Total Environ. 872, 162194. doi: 10.1016/j.scitotenv.2023.162194 36781130

[B22] Ibrahim M. Y. Hashim N. M. Mariod A. A. Mohan S. Abdulla M. A. Abdelwahab S. I.. (2016). α-mangostin from *Garcinia mangostana* Linn: an updated review of its pharmacological properties. Arabian J. Chem. 9, 317–329. doi: 10.1016/j.arabjc.2014.02.011

[B23] Iinuma M. Tosa H. Tanaka T. Asai F. Kobayashi Y. Shimano R.. (1996). Antibacterial activity of xanthones from guttiferaeous plants against methicillin-resistant *Staphylococcus aureus* . J. Pharm. Pharmacol. 48, 861–865. doi: 10.1111/j.2042-7158.1996.tb03988.x 8887739

[B24] Im A.-R. Kim Y. M. Chin Y.-W. Chae S. (2017). Protective effects of compounds from *Garcinia mangostana* l. (mangosteen) against UVB damage in HaCaT cells and hairless mice. Int. J. Mol. Med. 40, 1941–1949. doi: 10.3892/ijmm.2017.3188 29039482

[B25] Johnston A. M. (1998). Use of antimicrobial drugs in veterinary practice. BMJ 317, 665–667. doi: 10.1136/bmj.317.7159.665 9728002 PMC1113841

[B26] Jonsson K. McDevitt D. McGavin M. H. Patti J. M. Hook M. (1995). *Staphylococcus aureus* expresses a major histocompatibility complex class II analog. J. Biol. Chem. 270, 21457–21460. doi: 10.1074/jbc.270.37.21457 7545162

[B27] Joosten P. Ceccarelli D. Odent E. Sarrazin S. Graveland H. Van Gompel L. (2020). Antimicrobial usage and resistance in companion animals: a cross-sectional study in three European countries. Antibiotics 9, 87. doi: 10.3390/antibiotics9020087 32079072 PMC7175148

[B28] Jun S. H. Lee J. H. Choi C. W. Park T. I. Jung H. R. Cho J. W.. (2017). *Staphylococcus aureus*-derived membrane vesicles exacerbate skin inflammation in atopic dermatitis. Clin. Exp. Allergy 47, 85–96. doi: 10.1111/cea.12851 27910159

[B29] Jung H. A. Su B. N. Keller W. J. Mehta R. G. Kinghorn A. D. (2006). Antioxidant xanthones from the pericarp of *Garcinia mangostana* (Mangosteen). J. Agric. Food Chem. 54, 2077–2082. doi: 10.1021/jf052649z 16536578

[B30] Kim D. Langmead B. Salzberg S. L. (2015). HISAT: a fast spliced aligner with low memory requirements. Nat. Methods 12, 357–360. doi: 10.1038/nmeth.3317 25751142 PMC4655817

[B31] Koh J. J. Lin S. Aung T. T. Lim F. Zou H. Bai Y.. (2015). Amino acid modified xanthone derivatives: novel, highly promising membrane-active antimicrobials for multidrug-resistant gram-positive bacterial infections. J. Med. Chem. 58, 739–752. doi: 10.1021/jm501285x 25474410

[B32] Koh J. J. Qiu S. Zou H. Lakshminarayanan R. Li J. Zhou X.. (2013). Rapid bactericidal action of alpha-mangostin against MRSA as an outcome of membrane targeting. Biochim. Biophys. Acta 1828, 834–844. doi: 10.1016/j.bbamem.2012.09.004 22982495

[B33] Kreikemeyer B. McDevitt D. Podbielski A. (2002). The role of the map protein in *Staphylococcus aureus* matrix protein and eukaryotic cell adherence. Int. J. Med. Microbiol. 292, 283–295. doi: 10.1078/1438-4221-00212 12398219

[B34] Kugelberg E. Norström T. Petersen T. K. Duvold T. Andersson D. I. Hughes D. (2005). Establishment of a superficial skin infection model in mice by using *Staphylococcus aureus* and *Streptococcus pyogenes* . Antimicrob. Agents Chemother. 49, 3435–3441. doi: 10.1128/AAC.49.8.3435-3441.2005 16048958 PMC1196267

[B35] Lee L. Y. Miyamoto Y. J. McIntyre B. W. Höök M. McCrea K. W. McDevitt D.. (2002). The *Staphylococcus aureus* map protein is an immunomodulator that interferes with T cell–mediated responses. J. Clin. Invest. 110, 1461–1471. doi: 10.1172/JCI0216318 12438444 PMC151818

[B36] Leelapornpisid W. (2022). Efficacy of alpha-mangostin for antimicrobial activity against endodontopathogenic microorganisms in a multi-species bacterial-fungal biofilm model. Arch. Oral. Biol. 133, 105304. doi: 10.1016/j.archoralbio.2021.105304 34775269

[B37] Liu X. Shen J. Zhu K. (2022). Antibacterial activities of plant-derived xanthones. RSC Med. Chem. 13, 107–116. doi: 10.1039/D1MD00351H 35308024 PMC8864485

[B38] Nguyen P. T. Falsetta M. L. Hwang G. Gonzalez-Begne M. Koo H. (2014). Alpha-mangostin disrupts the development of *Streptococcus mutans* biofilms and facilitates its mechanical removal. PloS One 9, e111312. doi: 10.1371/journal.pone.0111312 25350668 PMC4211880

[B39] Palomino-Farfan J. A. Vega L. G. A. Espinoza S. Y. C. Magallanes S. G. Moreno J. J. S. (2021). Methicillin-resistant *Staphylococcus schleiferi* subspecies coagulans associated with otitis externa and pyoderma in dogs. Open Vet. J. 11, 364–369. doi: 10.5455/OVJ.2021.v11.i3.5 34722197 PMC8541725

[B40] Pedraza-Chaverri J. C´ ardenas-Rodríguez N. Orozco-Ibarra M. P´erez-Rojas J. M. (2008). Medicinal properties of mangosteen (*Garcinia mangostana*). Food Chem. Toxicol. 46, 3227–3239. doi: 10.1016/j.fct.2008.07.024 18725264

[B41] Pompilio A. De Nicola S. Crocetta V. Guarnieri S. Savini V. Carretto E.. (2015). New insights in *Staphylococcus pseudintermedius* pathogenicity: antibiotic-resistant biofilm formation by a human wound-associated strain. BMC Microbiol. 15, 109. doi: 10.1186/s12866-015-0449-x 25994406 PMC4440327

[B42] Rural Development Administration of Korea (2018). Analysis of reason for the visiting the veterinary hospitals in companion dogs. the report (Jeonju, Korea: Rural Development Administration). Available at: https://www.rda.go.kr/board/board.do?boardId=farmprmninfo&prgId=day_farmprmninfoEntry&currPage=1&dataNo=100000750287&mode=updateCnt&searchSDate=&searchEDate=&searchOrgDeptKey=org&searchOrgDeptVal=&searchKey=subject&searchVal=%EB%B0%98%EB%A0%A4%EA%B2%AC.

[B43] Sakagami Y. Iinuma M. Piyasena K. G. Dharmaratne H. R. (2005). Antibacterial activity of alpha-mangostin against vancomycin resistant enterococci (VRE) and synergism with antibiotics. Phytomedicine 12, 203–208. doi: 10.1016/j.phymed.2003.09.012 15830842

[B44] Silverman J. A. Perlmutter N. G. Shapiro H. M. (2003). Correlation of daptomycin bactericidal activity and membrane depolarization in *Staphylococcus aureus* . Antimicrob. Agents Chemother. 47, 2538–2544. doi: 10.1128/AAC.47.8.2538-2544.2003 12878516 PMC166110

[B45] Sivaranjani M. Leskinen K. Aravindraja C. Saavalainen P. Pandian S. K. Skurnik M.. (2019). Deciphering the antibacterial mode of action of alpha-mangostin on *Staphylococcus epidermidis* RP62A through an integrated transcriptomic and proteomic approach. Front. Microbiol. 10, 150. doi: 10.3389/fmicb.2019.00150 30787919 PMC6372523

[B46] Sivaranjani M. Prakash M. Gowrishankar S. Rathna J. Pandian S. K. Ravi A. V. (2017). *In vitro* activity of alpha-mangostin in killing and eradicating *Staphylococcus epidermidis* RP62A biofilms. Appl. Microbiol. Biotechnol. 101, 3349–3359. doi: 10.1007/s00253-017-8231-7 28343241

[B47] Tan Z. Shi Y. Xing B. Hou Y. Cui J. Jia S. (2019). The antimicrobial effects and mechanism of ϵ-poly-lysine against *Staphylococcus aureus* . Bioresour. Bioprocess 6, 11.

[B48] Tran V. A. Thi Vo T.-T. Nguyen M.-N. T. Duy Dat N. Doan V.-D. Nguyen T.-Q.. (2021). Novel α-mangostin derivatives from mangosteen (*Garcinia mangostana* l.) peel extract with antioxidant and anticancer potential. J. Chem. 2021, 9985604.

[B49] Van Boeckel T. P. Brower C. Gilbert M. Grenfell B. T. Levin S. A. Robinson T. P.. (2015). Global trends in antimicrobial use in food animals. Proc. Natl. Acad. Sci. U.S.A. 112, 5649–5654. doi: 10.1073/pnas.1503141112 25792457 PMC4426470

[B50] World Health Organization (2014). Antimicrobial resistance: global report on surveillance (World Health Organization). Available at: https://apps.who.int/iris/handle/10665/112642.

[B51] World Organisation for Animal Health (2016) The OIE strategy on antimicrobial resistance and the prudent use of antimicrobials. Available at: https://www.woah.org/fileadmin/Home/eng/Media_Center/docs/pdf/PortailAMR/EN_OIE-AMRstrategy.pdf.

[B52] Wu M. Hancock R. E. (1999). Interaction of the cyclic antimicrobial cationic peptide bactenecin with the outer and cytoplasmic membrane. J. Biol. Chem. 274, 29–35. doi: 10.1074/jbc.274.1.29 9867806

[B53] Xu N. Deng W. He G. Gan X. Gao S. Chen Y.. (2018). Alpha- and gamma-mangostins exhibit anti-acne activities via multiple mechanisms. Immunopharmacol. Immunotoxicol. 40, 415–422. doi: 10.1080/08923973.2018.1519831 30422030

[B54] Zaynab M. Fatima M. Abbas S. Sharif Y. Umair M. Zafar M. H.. (2018). Role of secondary metabolites in plant defense against pathogens. Microb. Pathog. 124, 198–202. doi: 10.1016/j.micpath.2018.08.034 30145251

[B55] Zou H. Koh J. J. Li J. Qiu S. Aung T. T. Lin H.. (2013). Design and synthesis of amphiphilic xanthone-based, membrane-targeting antimicrobials with improved membrane selectivity. J. Med. Chem. 56, 2359–2373. doi: 10.1021/jm301683j 23441632

