# Supplementary material for: Antimicrobial activity of α-mangostin against Staphylococcus species from companion animals in vitro and therapeutic potential of α-mangostin in skin diseases caused by S. pseudintermedius
Source: Front Cell Infect Microbiol. 2023 May 25;13:1203663. doi: 10.3389/fcimb.2023.1203663 (PMC10248440; doi:10.3389/fcimb.2023.1203663)
Supplement: Supplementary file 1 [file Presentation_1.pptx]

## Slide 1
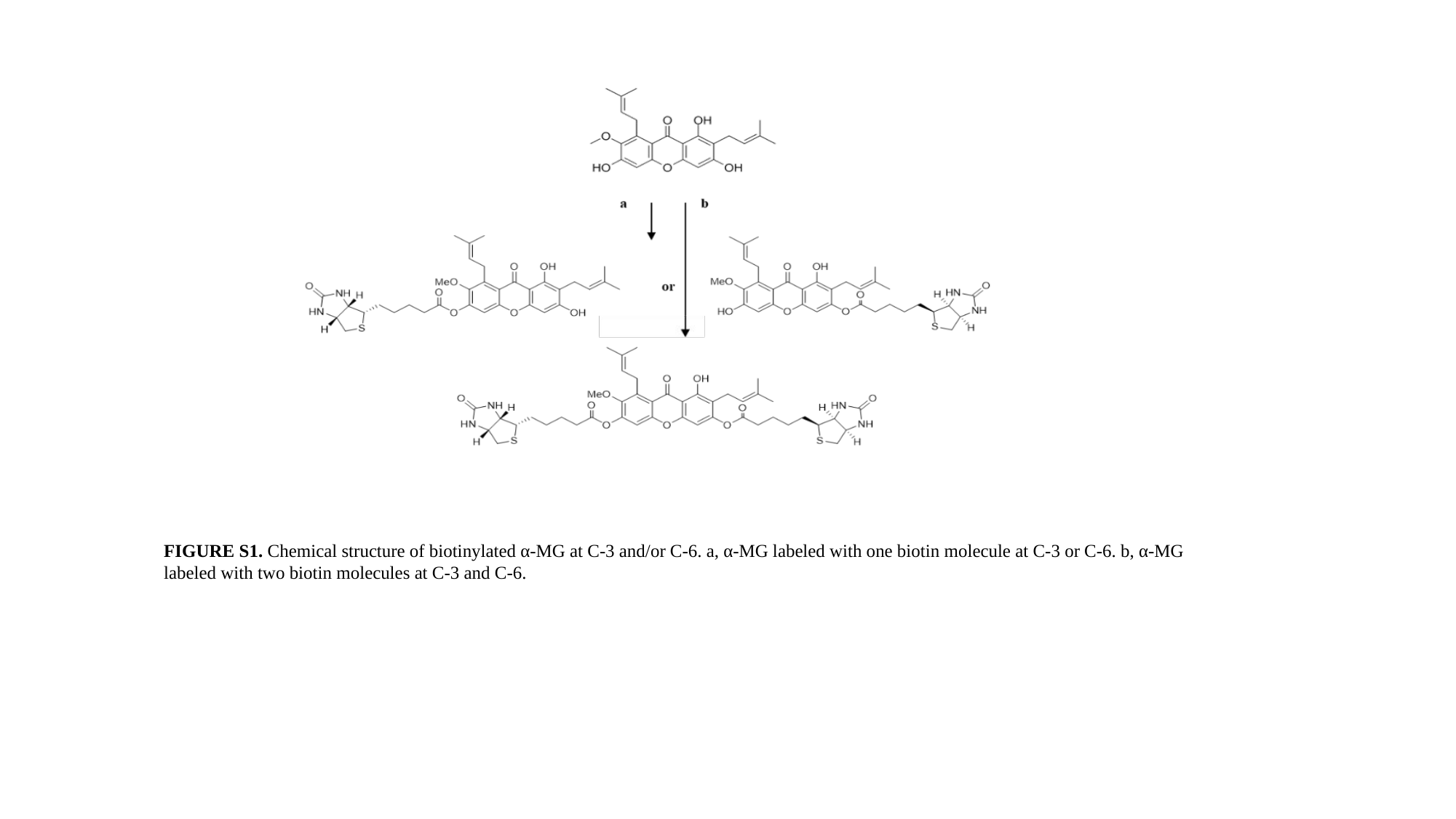

FIGURE S1. Chemical structure of biotinylated α-MG at C-3 and/or C-6. a, α-MG labeled with one biotin molecule at C-3 or C-6. b, α-MG labeled with two biotin molecules at C-3 and C-6.

## Slide 2
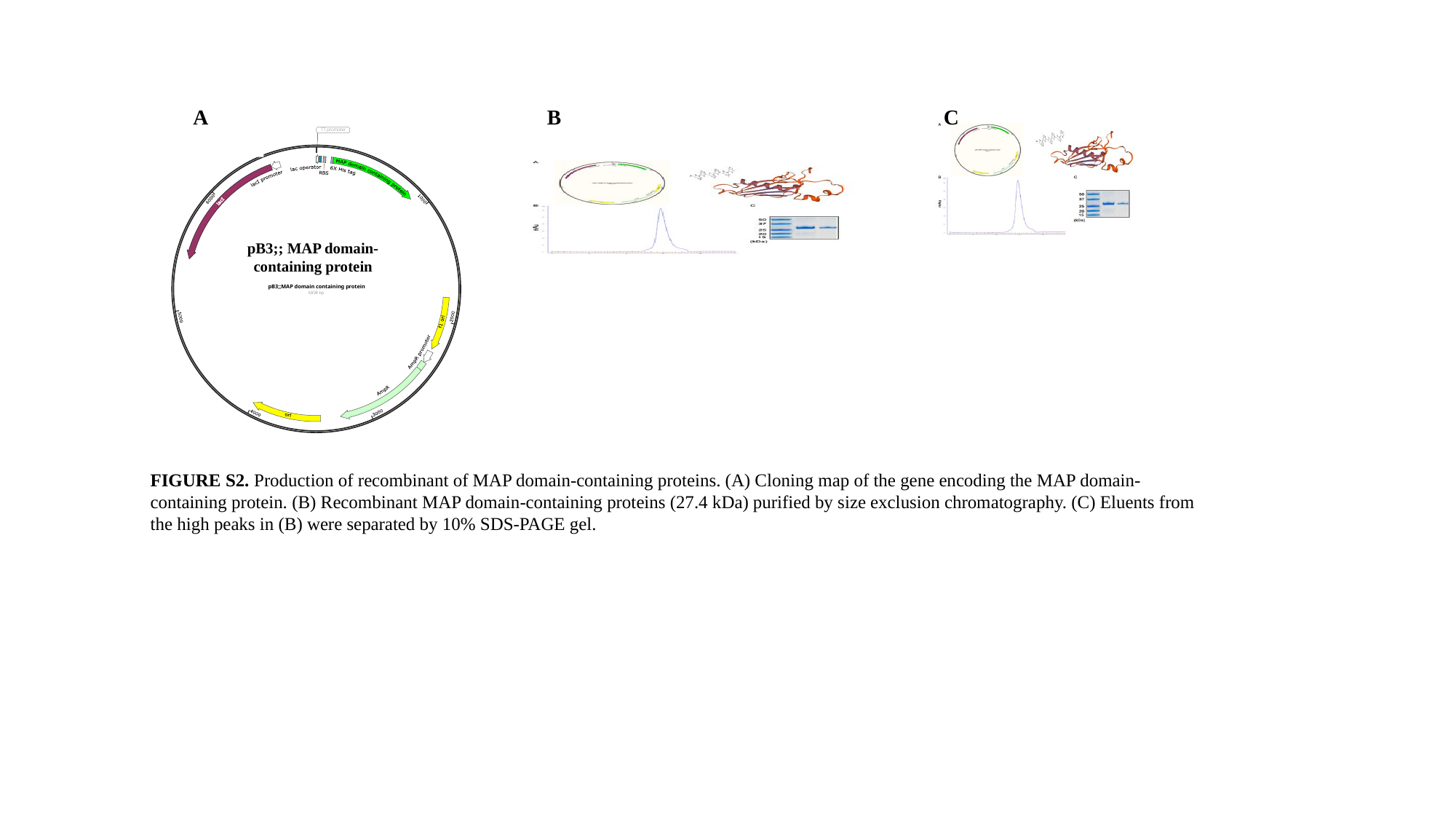

C
B
A
pB3;; MAP domain- containing protein
FIGURE S2. Production of recombinant of MAP domain-containing proteins. (A) Cloning map of the gene encoding the MAP domain-containing protein. (B) Recombinant MAP domain-containing proteins (27.4 kDa) purified by size exclusion chromatography. (C) Eluents from the high peaks in (B) were separated by 10% SDS-PAGE gel.

## Slide 3
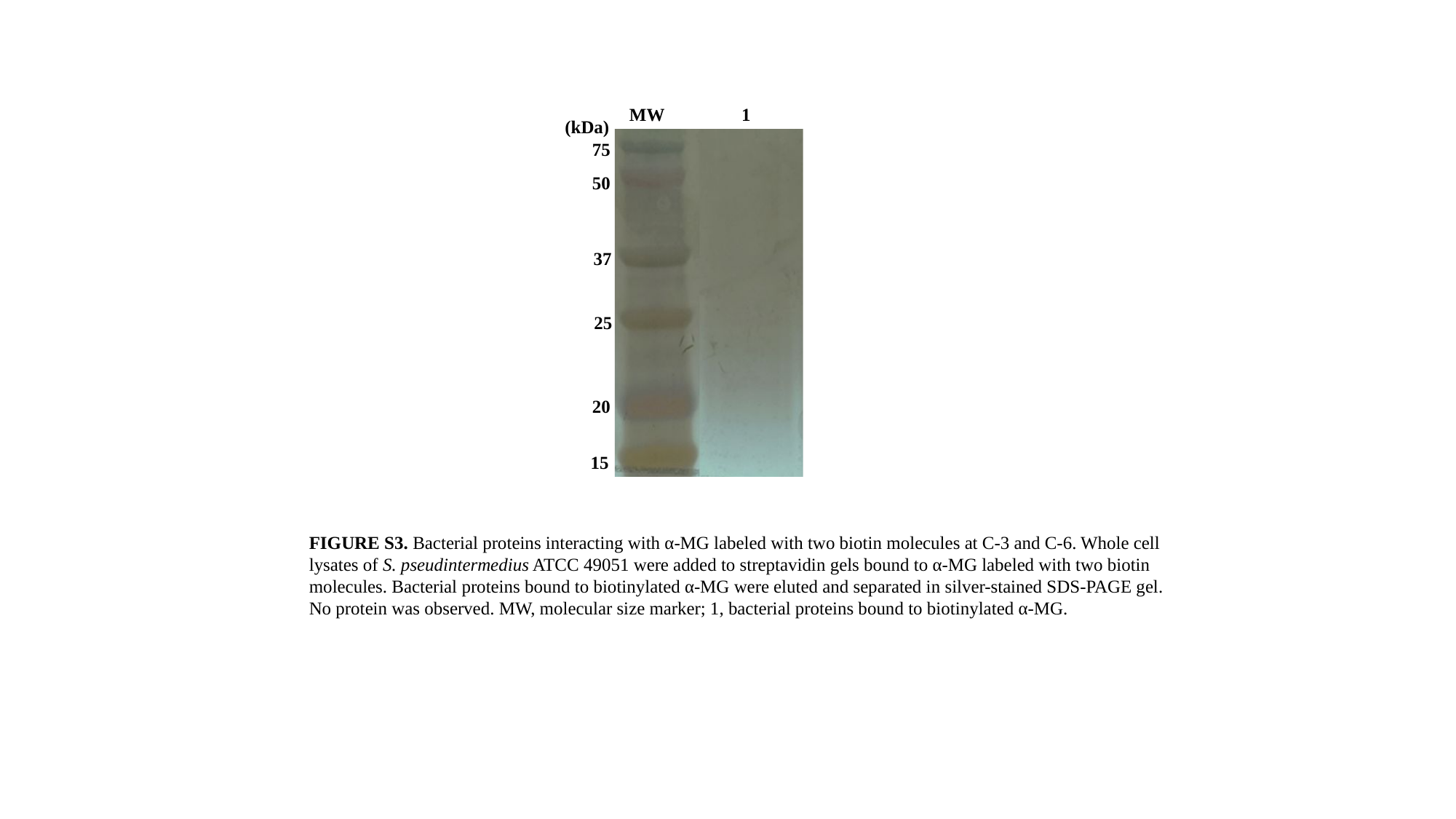

MW 1
(kDa)
75
50
37
25
20
15
FIGURE S3. Bacterial proteins interacting with α-MG labeled with two biotin molecules at C-3 and C-6. Whole cell lysates of S. pseudintermedius ATCC 49051 were added to streptavidin gels bound to α-MG labeled with two biotin molecules. Bacterial proteins bound to biotinylated α-MG were eluted and separated in silver-stained SDS-PAGE gel. No protein was observed. MW, molecular size marker; 1, bacterial proteins bound to biotinylated α-MG.

## Slide 4
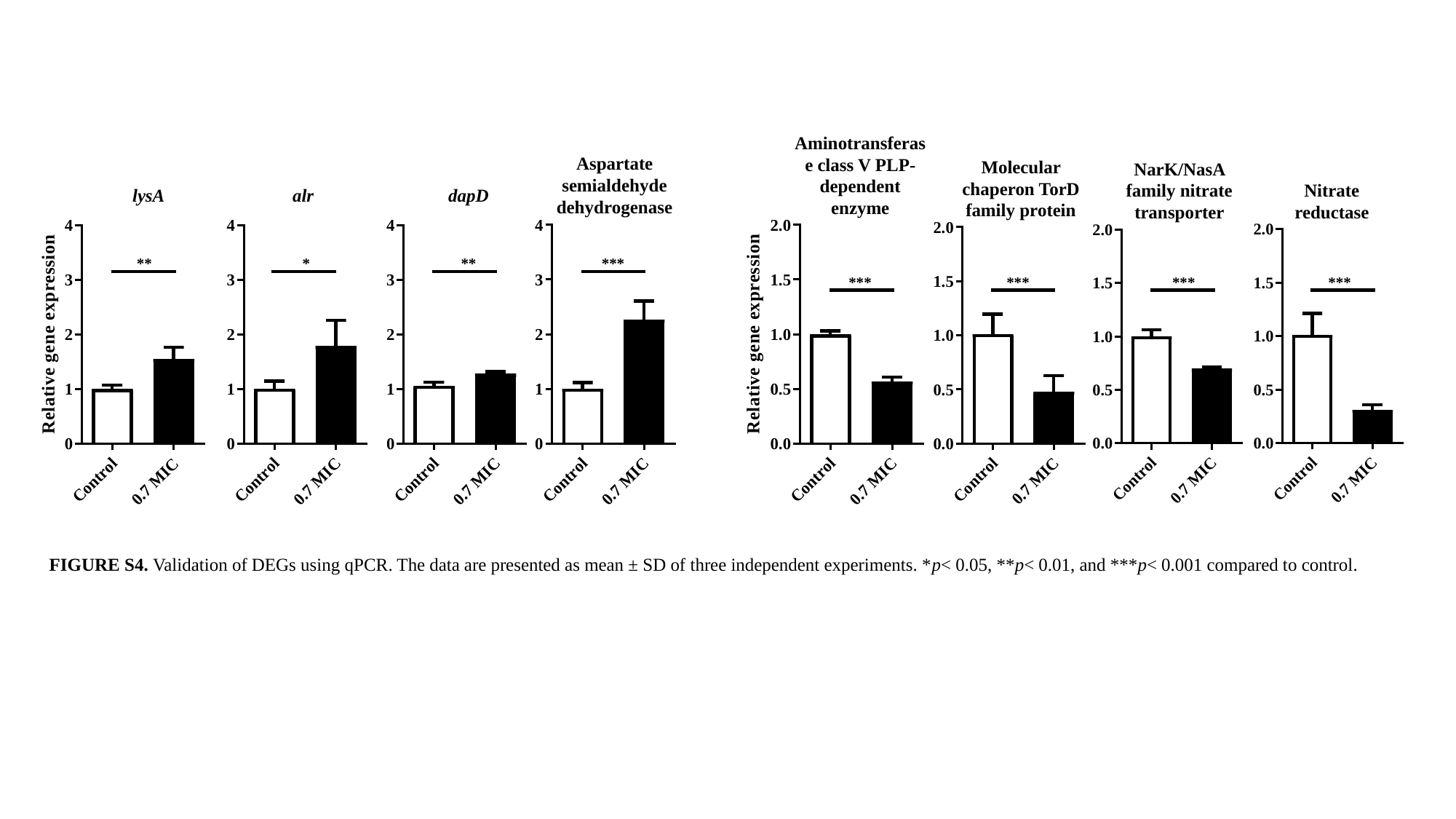

Aminotransferase class V PLP-dependent enzyme
Aspartate semialdehyde dehydrogenase
Molecular chaperon TorD family protein
NarK/NasA family nitrate transporter
Nitrate reductase
lysA
alr
dapD
**
***
**
*
***
***
***
***
FIGURE S4. Validation of DEGs using qPCR. The data are presented as mean ± SD of three independent experiments. *p< 0.05, **p< 0.01, and ***p< 0.001 compared to control.

## Slide 5
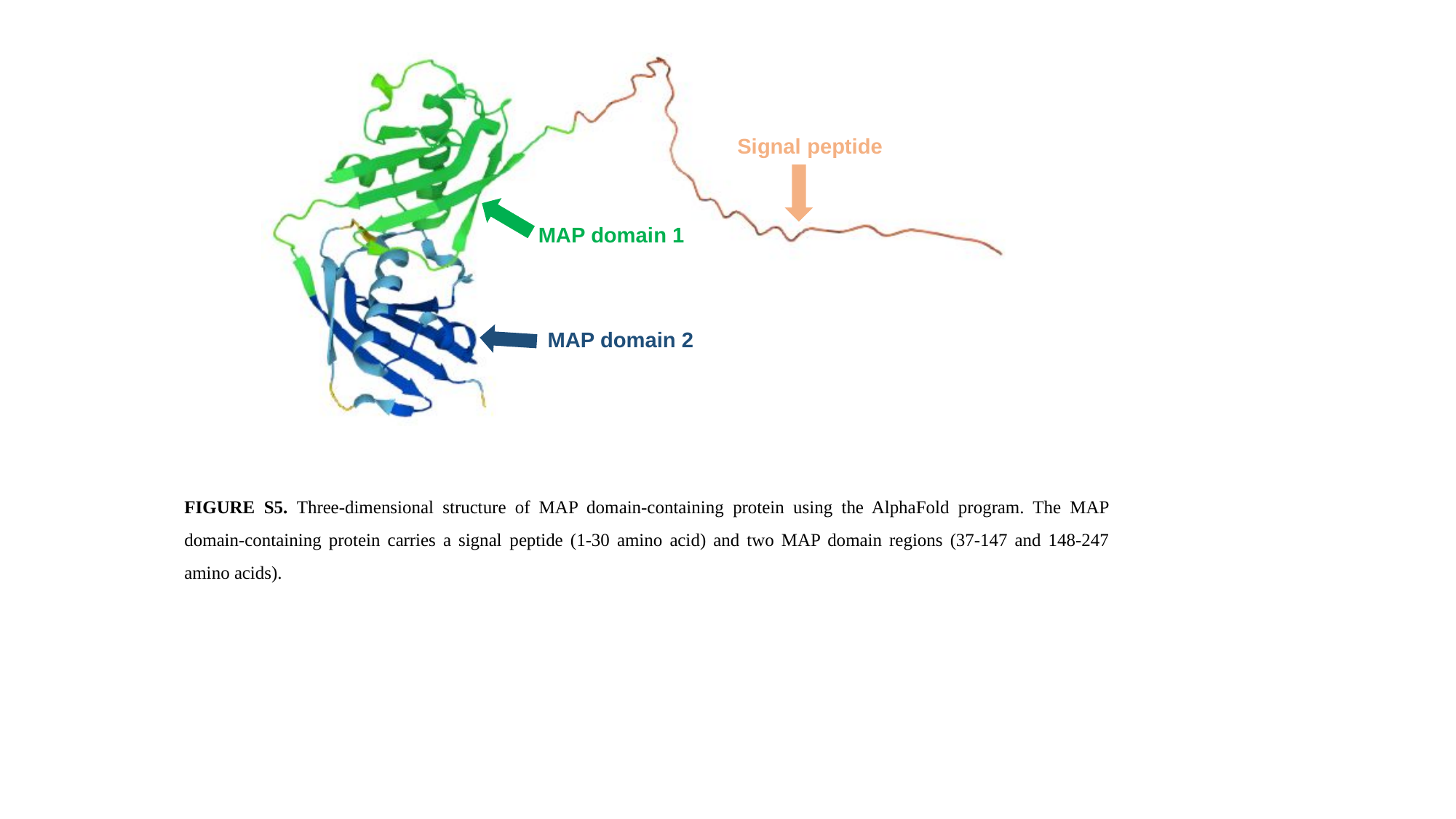

Signal peptide
MAP domain 1
MAP domain 2
FIGURE S5. Three-dimensional structure of MAP domain-containing protein using the AlphaFold program. The MAP domain-containing protein carries a signal peptide (1-30 amino acid) and two MAP domain regions (37-147 and 148-247 amino acids).

## Slide 6
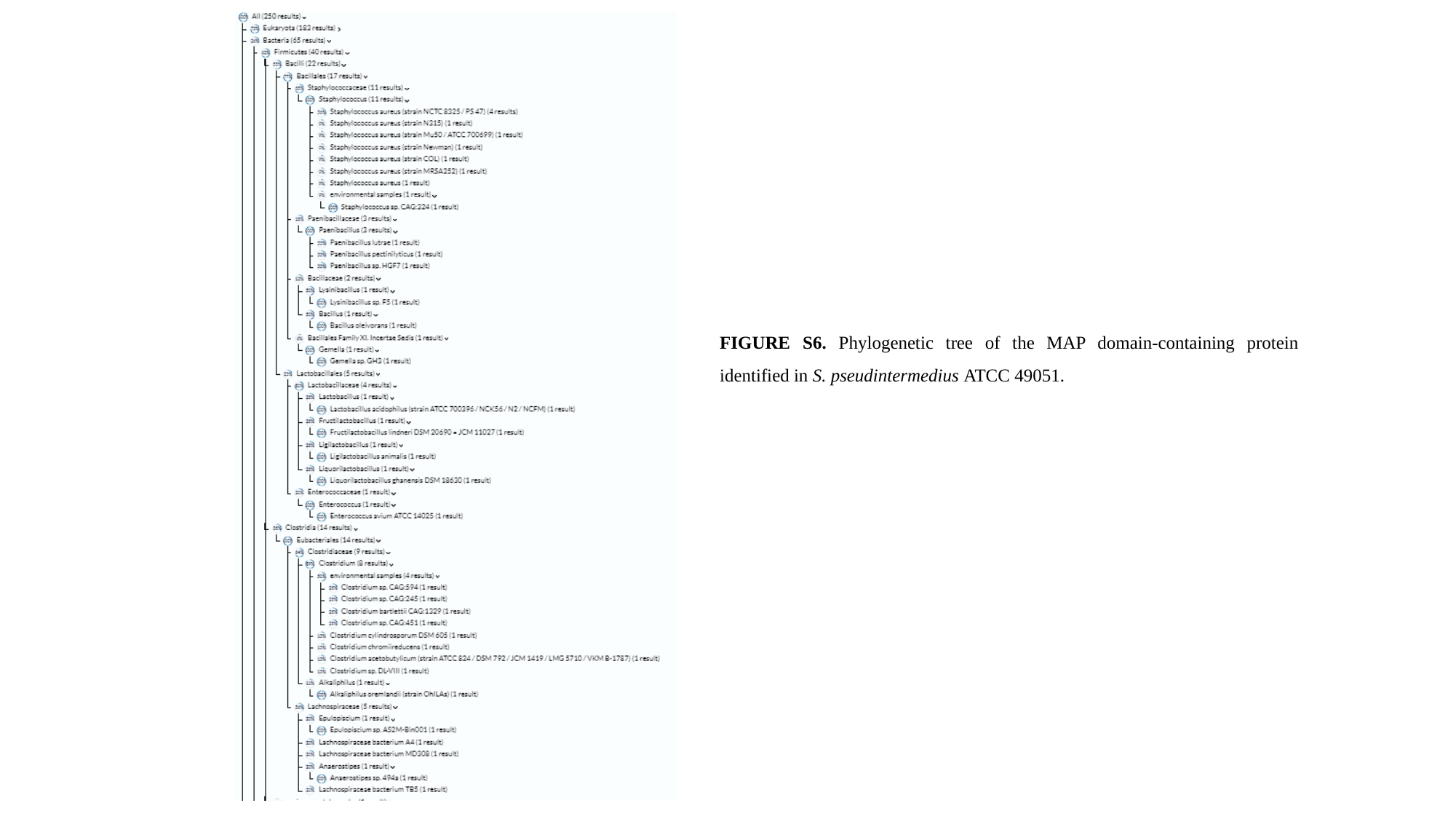

FIGURE S6. Phylogenetic tree of the MAP domain-containing protein identified in S. pseudintermedius ATCC 49051.
