# Supplementary material for: Antimicrobial activity of α-mangostin against Staphylococcus species from companion animals in vitro and therapeutic potential of α-mangostin in skin diseases caused by S. pseudintermedius
Source: Front Cell Infect Microbiol. 2023 May 25;13:1203663. doi: 10.3389/fcimb.2023.1203663 (PMC10248440; doi:10.3389/fcimb.2023.1203663)
Supplement: Supplementary file 2 [file Table_1.docx]

**Table S1.** Primers used for quantitative real-time PCR in this study

| Gene | Forward primer (5’ to 3’) | Reverse primer (5’ to 3’) | References |
| --- | --- | --- | --- |
| 16s rRNA | GGC GGC GTG CCT AAT ACA T | TCC GCC GCT AAC GTC AAA | This study |
| Diaminopimelate decarboxylase (*lysA*) | CCC CCA ACG CAT CCA TT | ACC ACT TTC AAG GGC ATA ACG A | This study |
| Alanine racemase (*alr*) | AGC TTG TCA CCT GGT TGC ATT | GGA CAT TTC GCA ATT GAA TCA C | This study |
| 2,3,4,5-tetrahydropyridine-2,6-dicarboxylate N acetyl transferase (*dapD*) | CGG CGA AAG TGA TCA AAC AA | TGA CGT AAC GCT GCG ACA AT | This study |
| Aspartate-semialdehyde dehydrogenase | GCT CAT TTT GCC CCA CTG TT | TCA TGC GCC ATT GAC TTG A | This study |
| Aminotransferase class V-fold PLP-dependent enzyme | GGC GAC ATT GAC TGC GAT TA | CGT TGC TTC AGA CAT TTG TGA GA | This study |
| Molecular charperone TorD family protein | CGG TCA AAT GTT GGC GAA GT | CAA CTC TGA AGC TGG CAT TTC A | This study |
| Nitrate reductase subunit beta (*narH*) | GGA GCG ACA AGC GAA GGA TA | TGG AAT GAC AAA ACG ATC TTC ATG | This study |
| NarK/NasA family nitrate transporter | TTC TTC CCA CCG CTT GTC A | ACA TAA CAA GAT GAA TGC GAA ATG A | This study |
| GAPDH | CAT CAC TGC CAC CCA GAA GAC TG | ATG CCA GTG AGC TTC CCG TTC AG | OriGene Technologies Inc.CAT#MP205604 |
| TNF-α | GGT GCC TAT GTC TCA GCC TCT T | GCC ATA GAA CTG ATG AGA GGG AG | OriGene Technologies Inc.CAT#MP217748 |
| IL-β | CCT GTC CTG CGT GTT GAA AGA | GGG AAC TGG GCA GAC TCA AA | OriGene Technologies Inc.CAT#MP206724 |
| IL-13 | AAC GGC AGC ATG GTA TGG AGT G | TGG GTC CTG TAG ATG GCA TTG C | OriGene Technologies Inc.CAT#MP206748 |
| IL-17A | CAG ACT ACC TCA ACC GTT CCA C | TCC AGC TTT CCC TCC GCA TTG A | OriGene Technologies Inc.CAT#MP206759 |
| IFN- γ | CAG CAA CAG CAA GGC GAA AAA GG | TTT CCG CTT CCT GAG GCT GGA T | OriGene Technologies Inc.CAT#MP206663 |
